# Gender differences in attitudes toward academic effort and boys’ underachievement: A large-scale international study

**DOI:** 10.1093/pnasnexus/pgag316

**Published:** 2026-10-13

**Authors:** Clotilde Napp

**Affiliations:** CNRS, UMR7088, 75016 Paris, France; Université Paris-Dauphine, PSL Research University, Place du Maréchal de Lattre de Tassigny, 75016 Paris, France

**Keywords:** boys’ crisis, gender differences in academic effort, gender equality paradox, gender stereotypes, cross-national comparison

## Abstract

In most developed countries, boys underperform academically compared with girls. One possible explanation lies in boys’ more negative attitudes toward school and learning. Using data from 750,000 15-year olds across 61 countries, we investigate gender gaps in attitudes toward academic effort, including students’ beliefs about the importance of trying hard in school. Boys consistently report less positive attitudes than girls, regardless of socioeconomic status, family structure, or grade repetition. These gender gaps are observed in nearly all countries and are larger in more developed societies. At the individual level, attitudes toward effort are linked to academic achievement, particularly in reading. At the national level, countries with larger gender gaps in effort-related attitudes tend to have more boys among low performers and fewer boys in tertiary education, especially in the arts and humanities, but not in STEM (science, technology, engineering, and mathematics) fields. Finally, across countries, these gaps are associated with gender differences in ability beliefs and parental support and with the strength of gender stereotypes, pointing to the role of gender norms in shaping educational attitudes. These findings suggest that cultural and psychological factors may play a key role in shaping gender disparities in education.

Significance statement Boys are nowadays outperformed by girls in terms of academic achievement in most Western countries. They get lower grades, drop out of school more often, and pursue higher education less frequently than girls. Their lower academic effort has been advanced as a possible explanation for this “boys’ crisis.” We propose an international analysis of the gender differences in attitudes toward academic effort based on data from 750,000 15-year olds across 61 countries. This analysis sheds light on these gender differences—their variation across socioeconomic groups and countries, their possible determinants and consequences, and, in particular, their relation to gender differences in beliefs about ability (such as growth mindset), to gender stereotypes, and to the “boys’ crisis.”

## Introduction

In Western countries, growing concern has emerged over what is often referred to as the “boys’ crisis” in education. Boys increasingly underperform relative to girls, earning lower grades (for an international meta-analysis, see (1)), dropping out of school at higher rates (for an overview, see (2)), and being underrepresented in higher education institutions (3). In the United States, for example, high school grade point averages are now significantly lower for boys than for girls (4) and almost 40% of females from the 1985 birth cohort had a college degree by age 30, compared with 30% of males (5)—a reversal of historical patterns of male overrepresentation in tertiary education. Across Organization for Economic Co-operation and Development (OECD) countries, women constituted 54% of new entrants to bachelor's degrees in 2017 (3). Understanding the factors driving these gender disparities is critical for informing educational policy and promoting equity.

Research suggests that boys exhibit less positive attitudes toward school and academic effort compared with girls, which may explain the so-called boys’ crisis (6–11). Theoretical models of academic learning and achievement identify students’ academic engagement and attitudes toward school effort as key predictors of academic success (6, 7, 9, 12, 13). Empirical studies, primarily conducted in the United States and other economically developed countries, indicate that girls report more positive attitudes toward academic effort than boys and that these more positive attitudes may explain gender differences in academic achievement (7–9, 11, 14–17).

Recent literature further highlights the possible role of gender norms in shaping boys’ and girls’ attitudes toward school, with boys being expected to conform to identities that are less oriented toward academic effort (6–8, 16, 18–22).

Most of this evidence, however, comes from single-country studies. A large-scale cross-national analysis of gender differences in attitudes toward academic effort would allow one to examine, together, how these differences relate to gender gaps in academic achievement and to the gender norms and broader cultural factors that may shape them—offering a more complete understanding of the mechanisms at play.

In the present work, we use large-scale, cross-national data from the Programme for International Student Assessment (PISA 2012 and PISA 2018), comprising over 750,000 observations across 61 countries, to provide a large-scale cross-national analysis of gender gaps in attitudes toward academic effort. We measure effort-related attitudes using students’ self-reported valuation of trying hard at school, and we confirm the robustness of our results using a PISA index of attitudes toward learning activities. Our analysis makes three main contributions. First, we establish that girls report more positive attitudes toward academic effort than boys consistently and robustly—in nearly all countries and across subgroups defined by socioeconomic status, family structure, and grade repetition. Second, we show that these effort-related gaps are tightly linked to the “boys’ crisis”: at the individual level, effort attitudes are associated with achievement, most strongly in reading; at the country level, larger gaps coincide with boys’ lower relative performance and their underrepresentation in tertiary education—though, notably, not in STEM (science, technology, engineering, and mathematics) fields. Third, we show that these gaps are larger in more developed countries and that their cross-national variation co-varies with sociocultural factors—gender gaps in ability beliefs and in parental support for effort, and the strength of gender stereotypes—pointing to the role of gender norms in shaping educational attitudes. One interpretation, which we develop in the discussion, is that these patterns partly reflect a cultural stereotype associating talent and brilliance with boys (23, 24) and effort and diligence with girls (25, 26), though we treat this as a tentative reading rather than an established mechanism.

Our work is related to, but distinct from, two recent strands of research. A first, large body of cross-national work documents a “gender equality paradox”—in several domains, gender differences are larger, not smaller, in more developed and gender-equal countries. Much of this work concerns STEM, focusing on girls’ relative academic strengths and their underrepresentation in STEM fields (27–31) or on care-oriented aspirations (32). Our focus differs: we study overall academic standing and progression (performance across domains, access to tertiary education) rather than the relative position of one domain—be it girls’ underrepresentation in STEM or boys’ in care-oriented fields—and we do so through attitudes toward effort rather than cognitive strengths or field-specific aspirations. We share the cross-national framework and the paradox of widening gaps but consider a different phenomenon—the “boys’ crisis.” A second strand investigates this crisis more directly, exploring explanations that range from school environments and teacher–student relationships to motivational dynamics. For instance, boys have been found to report more positive perceptions of their learning environment than girls despite lower achievement (33), suggesting that their underachievement does not stem from a less favorable experience of school itself. We complement this work by examining an underexamined dimension—not how boys perceive school, but the value they place on academic effort.

The Results section starts by introducing our measures of effort-related attitudes and by quantifying the gender gap, examining its variation across individual characteristics and countries. It then explores the relationship between effort-related attitudes, academic performance, and educational pathways. Finally, it investigates potential sociocultural determinants of the gender gap. The Discussion section considers the implications of our findings for understanding and addressing gender disparities in education, as well as the limitations of our analysis.

## Results

Our primary measure of effort-related attitudes is students’ agreement that “Trying hard at school is important” (THI); we confirm all results using the PISA index of attitudes toward learning activities (ATTLNACT). Both are available in PISA 2012 and PISA 2018 (the only waves including these measures). THI uses response options from “strongly agree” to “strongly disagree,” reverse-coded so that higher values indicate more positive attitudes (764,461 students from 61 countries); ATTLNACT is built from four school-related statements (762,935 students). For clarity, the main text reports THI; all results replicate using ATTLNACT and are reported in the Supplementary material. Variables, data sources, and methods are detailed in “Materials and methods” and the Supplementary material.

## Boys value academic effort less than girls, particularly in more developed countries

Across the full sample, boys report less positive effort-related attitudes than girls: the average gender gap is 0.20 SD (Table S1A). This pattern is highly general—gaps favoring girls are positive and significant at the 1% level in 60 of 61 countries (Korea at 5%), ranging from 0.05 SD (Korea) to 0.36 SD (Norway)—and broadly consistent across the 2012 and 2018 cycles (Table S1A).

The gap is robust to individual controls: adding socioeconomic status, age, parental education, grade level, and grade repetition does not materially change it, nor does adjusting for school belonging and truancy, nor allowing these covariates to interact with gender (Table S1B). It is also present across student subgroups—repeaters and nonrepeaters, grade levels, parental education, and family structures with only small differences by single-parent status (Table S1C).

Grouping the 61 countries into eight cultural regions reveals systematic variation in magnitude while preserving the same direction everywhere (Table S1C): gaps are smallest in East/Southeast Asia (0.13 SD) and Latin America (0.15 SD), largest in the Nordics and Middle East/Central Asia (0.28 SD), with other regions in between. This cultural patterning is accompanied by modest spatial autocorrelation, driven primarily by the East/Southeast Asia region; all cross-country results are re-estimated with spatial error models (Appendix E).

We then examine how the gap varies with country development for two reasons: its connection to the Gender Equality Paradox—the finding that in many domains gender differences are larger, not smaller, in more developed countries (27, 34)—and the fact that the boys’ crisis has been documented mainly in developed countries. Country-level gaps are larger in more developed countries: they are positively and significantly associated with all indicators of development we consider (Economic Social and Cultural Status [ESCS]: *R* = 0.55, Gross Domestic Product [GDP] *R* = 0.42, Human Development Index *R* = 0.40; Fig. 1, Table S1D), as well as with measures of gender equality and individualism (Table S1D). Similar patterns hold for historical indicators (e.g. GDP in 1980; Table S1D), suggesting historical roots.

**Figure 1:**
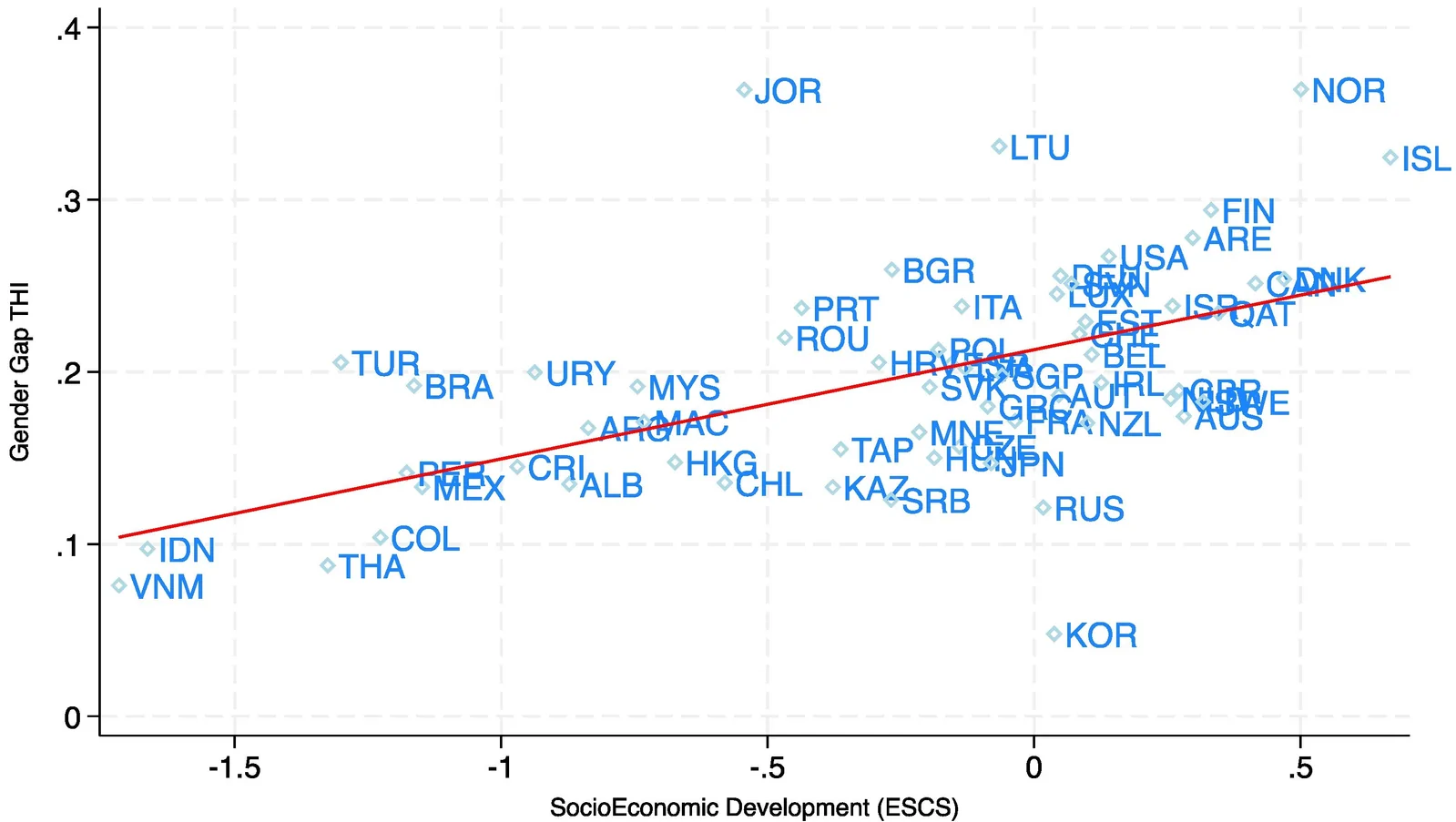
The gender equality paradox for attitudes toward academic effort. The figure presents national gender gaps in attitudes toward academic effort based on data from the PISA 2012 and PISA 2018 surveys, shown as a function of each country’s level of socioeconomic development, measured using the PISA ESCS index (averaged over PISA 2012 and PISA 2018). Attitudes toward academic effort are captured by students’ agreement with PISA statement “Trying hard at school is important” (THI). A positive gender gap in THI indicates that girls have more positive attitudes than boys toward trying hard at school. All variables and data sources are described in Appendices A and B. Country codes from ISO3166-1 standard.

We subjected this association to a series of robustness checks addressing recent methodological critiques of cross-national gender-gap research (28, 35, 36). Cross-country variation is substantial rather than trivial (Cochran's *Q* = 487, *P* < 0.001; *I*^2^ = 87.7%); the association is robust across alternative specifications—including clustering, hierarchical (multilevel) linear models that account for the nesting of students within countries, nonstandardized variables, and unweighted estimates—and is not driven by individual-level confounds (Table S1E). All results replicate using the ATTLNACT index (Table S1A–E).

## Gender differences in attitudes toward academic effort are related to gender differences in academic achievement and educational pathways

### Academic achievement

We measure performance using PISA scores in mathematics, reading, and science, a composite general-performance index (the average of the three; Appendix A), and the probability of being a low performer (bottom half of the national distribution within a given cycle, for general and for reading performance). At the individual level, effort-related attitudes are positively associated with achievement (Table S2A): students reporting more positive attitudes have higher scores in mathematics, reading, and science—most strongly in reading (Table S2A, panel iii)—and are less likely to be low performers. These relationships hold for both boys and girls; in reading, the association is larger for boys (Table S2A, panels i and ii).

At the country level, gender gaps in effort-related attitudes are associated with gender gaps in performance in the expected direction (Tables 1 and S2B). Countries where boys’ effort-related attitudes lag further behind girls’ are those where boys perform relatively worse and are overrepresented among low performers, most strongly for reading: a 1-SD increase in the gender gap in THI is associated with a 0.35-SD decrease in the gender gap (boys - girls [B - G]) in math score, a 0.46-SD increase in the gender gap (girls - boys [G - B]) in reading score, and a 0.47-SD increase in the gender gap (B - G) in the likelihood of being a low performer in reading.

**Table 1:** Gender differences in educational outcomes and gender differences in attitudes toward academic effort (country-level OLS).

Variable	Boys’ reading score relative to girls	Boys’ math score relative to girls	Boys’ likelihood of being low performer relative to girls	Share males tertiary education	Share males in tertiary humanities	Share males in tertiary STEM
Gender gap THI (G - B)	−0.463***	−0.350**	0.407***	−0.512**	−0.423**	−0.196
	(0.144)	(0.143)	(0.151)	(0.205)	(0.177)	(0.207)
Constant	4.37e-10	−1.72e-09	3.97e-09	0.119	0.0731	0.0385
	(0.114)	(0.121)	(0.118)	(0.165)	(0.150)	(0.166)
Observations	61	61	61	39	41	40
*R* ^2^	0.174	0.093	0.141	0.227	0.153	0.033

The table presents estimates at the country level of linear regressions of gender differences in educational outcomes on the gender gap (G - B) in attitudes toward trying hard at school (THI). Positive gender gaps in THI represent more positive attitudes toward trying hard at school for girls than for boys. The first columns consider gender gaps (B - G) in academic performance, where academic performance is captured by PISA score in reading (column 1), in math (column 2), and by the likelihood of being a low performer (i.e. performing below average in their country in the given PISA cycle, column 3). Column 4 considers the share of males among students aged 19–22 enrolled in tertiary education, while the last columns consider the share of males among students enrolled in tertiary education in the specific fields of arts and humanities (column 5) and STEM (column 6). All variables and data sources are described in Appendices A and B. The model is described in Appendix C (model 3b). Robust SEs are given in parentheses. ***P < 0.01, **P < 0.05, *P < 0.10.

### Tertiary education and expected years of schooling

Using OECD data on the share of 19- to 22-year olds enrolled in tertiary education who are male (Appendix B), we find that this share is below 50% in all countries except Korea (average: 44%). The share of males in tertiary education is lower in countries with larger gender gaps in effort-related attitudes (Tables 1 and S2C, Fig. 2; *R* = −0.51 for THI, −0.43 including Israel): where boys’ effort-related attitudes are closer to girls’, the male share in tertiary education is correspondingly higher. A similar association holds for gender gaps in expected years of schooling (EYS), a standard cross-national indicator of educational access (a 1-SD increase in the THI gap being associated with a 0.35-SD increase in the gap in EYS; Table S2C).

**Figure 2:**
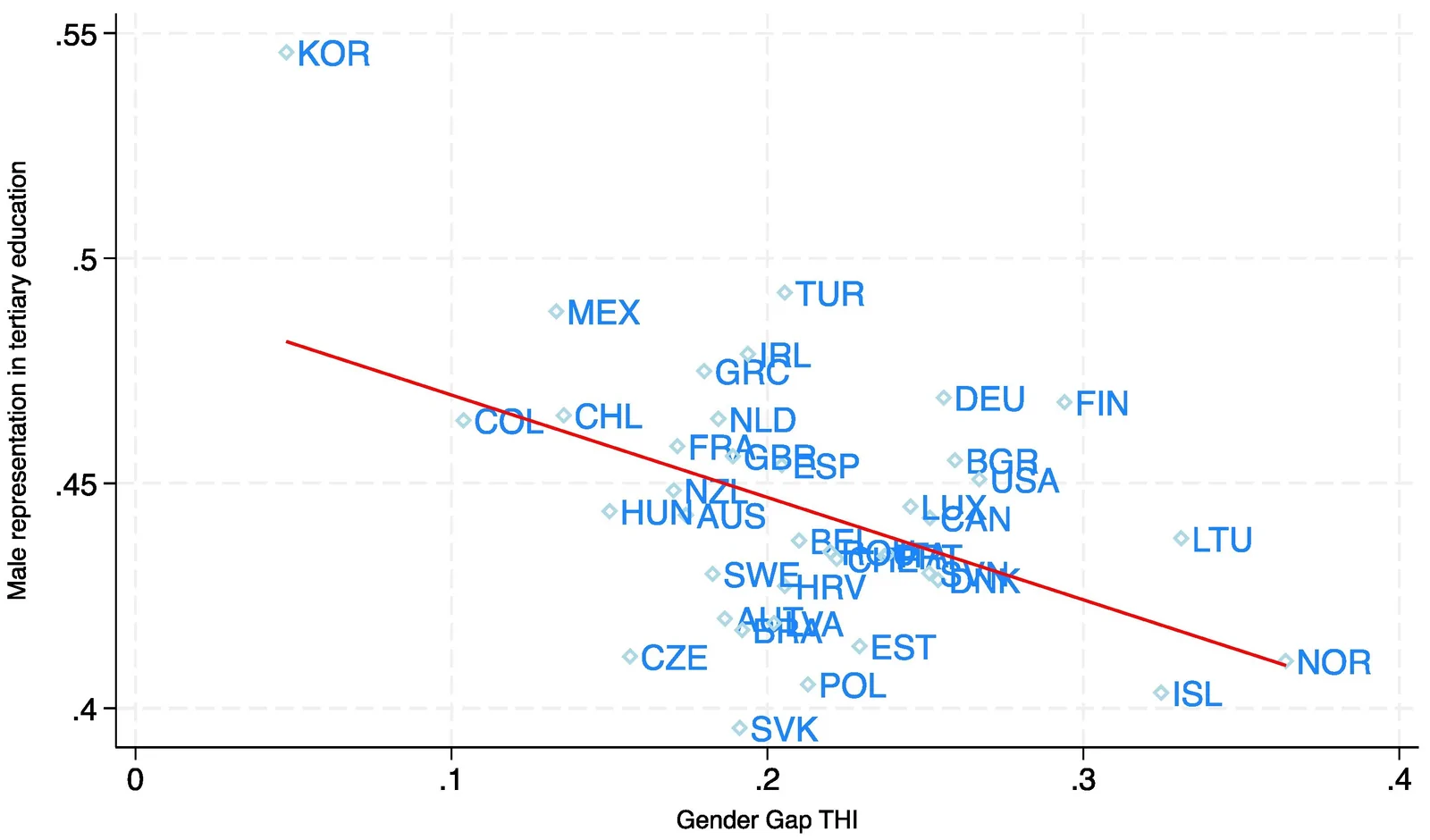
Gender gaps in educational outcomes and gender gaps in attitudes toward academic effort. The figure presents the national levels of male (under)representation in tertiary education (share of males among students enrolled in tertiary education, OECD data, 2017) as a function of the gender gap in attitudes toward academic effort, captured by students’ agreement with PISA statement “Trying hard at school is important” (THI) relying on PISA 2012 and PISA 2018 surveys. A positive gender gap in THI indicates that girls have more positive attitudes toward trying hard at school. Variables and data sources are described in Appendices A and B. Country codes from ISO3166-1 standard.

### Field of study and occupational expectations

These associations differ by field (Tables 1 and S2D). The share of males in arts and humanities is strongly (negatively) related to effort-attitude gaps (*R* = −0.42): male underrepresentation in these fields is stronger in countries with larger gender gaps in effort. In contrast, the association is not significant for STEM in absolute terms and is even positive when STEM is considered relative to other fields (Table S2D). Consistently, PISA 2012 data show no weaker male intentions to pursue math studies or careers in countries with larger effort-attitude gaps—if anything, slightly stronger (Table S2D). Finally, girls are more likely than boys in every country to expect a health-related career by age 30, and countries with larger effort-attitude gaps show greater female overrepresentation in expected health occupations (*R* = 0.34; Table S2D).

In sum, countries where boys report more negative effort-related attitudes relative to girls are those where boys underperform—particularly in reading—and are more underrepresented in tertiary education, especially in arts/humanities and health-related pathways, but not in STEM or math-oriented ones. All results replicate using the ATTLNACT index (Table S2A–D).

## Gender differences in attitudes toward academic effort are associated with gender gaps in ability beliefs and gender stereotypes

For many boys, trying hard at school is seen as inappropriate or contrary to masculine identity, and academic effort more broadly is coded as feminine (7, 14, 16, 19–21, 37, 38); in (16), boys were 1.75 times as likely as girls to report they would be unpopular, and 1.50 times as likely to be made fun of for trying hard at school. This suggests that the gender gap in effort-related attitudes may itself be an expression of broader gender norms. We probe this by asking whether, across countries, this gap co-varies with other gender differences that reflect such norms—gender gaps in students’ ability beliefs (growth mindset and fear of lacking talent) and in perceived parental support—and with national gender stereotypes (Table 2).

**Table 2:** Gender gaps in attitudes toward academic effort, ability beliefs, and gender stereotypes (country-level analysis).

	Dependent variable is the gender gap in THI		
Explanatory variable	Coefficient (SE)	Obs.	*R* ^2^
Gap in growth mindset	0.419*** (0.100)	60	0.195
Gap in fear of lacking talent	0.297** (0.140)	59	0.097
Gap in parental support	0.279* (0.141)	59	0.077
Implicit career-family stereotypes	0.434*** (0.123)	53	0.194
Communality stereotypes	0.437*** (0.134)	42	0.217

The table presents estimates at the country level of linear regressions of the gender gap in attitudes toward academic effort on gender differences in ability beliefs, parental support, and on gender stereotypes; each row reports a separate regression. Attitudes toward academic effort are measured by attitudes toward trying hard at school (THI). Positive gender gaps in THI represent more positive attitudes toward trying hard at school for girls than for boys. The first three rows consider gender differences (G - B) in growth mindset, in attribution of failure to lack of talent, and in perceived parental support, based on PISA data (positive values indicate higher levels for girls). The last two rows consider country-level implicit career-family stereotypes, from Project Implicit (39), and prescriptive communality stereotypes, from (40, 41) (positive values indicate stronger association of career with men and family with women and higher desirability of communal traits for women than men, respectively). All variables are described in Appendices A and B and standardized on the regression sample. The model is described in Appendix C (model 3a). Robust SEs are given in parentheses. ***P < 0.01, **P < 0.05, *P < 0.10.

### Gender differences in ability beliefs

We consider two distinct facets of students’ beliefs about ability (42, 43): their growth mindset and their fear of lacking talent when facing failure.

A growth mindset is the belief that ability is malleable and can improve through effort, rather than being fixed. PISA 2018 asks students whether they agree that “Your intelligence is something about you that you can’t change very much”; we reverse-code this item so that higher values indicate a stronger growth mindset (Appendix B). At the individual level, growth mindset is positively associated with effort-related attitudes (Table S3A), consistent with prior research (11, 44–46). At the country level, the gender gap in growth mindset is positively associated with the gap in effort-related attitudes (*R* = 0.42; Tables 2 and S3B, Figs. S1 and S2). Since believing that ability can be improved through effort makes effort worthwhile, the two gaps are expected to move together: countries where the gender gap in growth mindset (G - B) is larger are indeed those where the effort gap (G - B) is larger.

Fear of lacking talent is captured by the PISA 2018 item “When I am failing, I am afraid I might not have enough talent,” which girls endorse more than boys (gap = 0.38 SD). This gap may reflect internalization of the stereotype that boys are talented and girls are not ((47); see (23, 24) on the brilliance stereotype). At the individual level, fear of lacking talent is positively related to effort-related attitudes (Table S3A); one interpretation is that fearing a lack of talent leads students to compensate through academic effort, whereas relying on presumed talent leads them to value effort less. At the country level, the gap in fear of lacking talent is also positively, though weakly, associated with the effort gap (*R* = 0.30; Tables 2 and S3B).

### Gender differences in parental support

If effort is more strongly associated with femininity, parents may support it more in girls. PISA 2018 asks students how much they agree with the statement “My parents support my educational efforts and achievements”; girls are more likely than boys to strongly agree, in all countries. At the country level, this gender gap is modestly associated with the gap in effort-related attitudes (*R* = 0.28; Tables 2 and S3B).

### Gender stereotypes

Gender stereotypes traditionally associate men and women with distinct traits—agency vs. communality, career vs. family, competence vs. warmth (48, 49). These deeply rooted cultural markers of normative gender expectations vary across countries and can shape the development of gendered aspirations and attitudes toward learning and academic effort. According to gender socialization theory, the gender gap in valuing academic effort is expected to be wider in countries where such stereotypes are stronger and where masculine and feminine identities are more sharply differentiated. Furthermore, because effort and diligence are themselves stereotypically associated with women (25, 26), we expect this gap to be most strongly related to the communal dimension of gender stereotypes.

Using implicit career-family stereotypes from Project Implicit (39), we find larger effort gaps in countries that more strongly associate career with men and family with women (*R* = 0.43; Tables 2 and S3B, Fig. S3). Using cross-cultural measures of prescriptive stereotypes (40, 41), we find that boys’ effort-related attitudes lag further behind girls’ in countries with stronger prescriptive communality stereotypes—that is, where communal traits are more strongly prescribed for women than for men (*R* = 0.44; Tables 2 and S3B, Fig. S4). In contrast, prescriptive agentic stereotypes show no significant relation (Table S3B). All results are replicated using the ATTLNACT index (Table S3A and B).

## Discussion

Based on PISA data with more than 750,000 observations from 61 different countries, we have shown that girls have more positive attitudes toward academic effort than boys. These gender differences are evident regardless of students’ socioeconomic status, family structure, or parental education level, and they are especially prevalent in more economically developed countries (a phenomenon we refer to as the Gender Equality Paradox for academic effort). Countries with the largest gender differences in these attitudes also have the greatest gender disparities in academic performances, the strongest male underrepresentation in higher education and the largest gender gaps in EYS, though this underrepresentation appears limited to certain fields, such as the humanities, and is not observed in STEM. Our analysis further shows that the effort gap is related to broader gender norms: it is larger where other gender differences that reflect such norms are more marked—in ability beliefs (growth mindset and fear of lacking talent) and in parental support—and where national gender stereotypes (communality, career vs. family) are stronger.

These findings speak directly to the “boys’ crisis”: the gender gap in academic effort is present across all groups we considered, regardless of personal characteristics, and is meaningfully associated with gender disparities in academic achievement—which raises concern.

The fact that this gap is more pronounced in economically developed countries reinforces the idea of the “Gender Equality Paradox,” whereby more economically and more gender-equal societies tend to exhibit greater gender differentiation in preferences or behaviors. This highlights the complexity of the mechanisms at play: formal equality of opportunity does not necessarily translate into convergence in attitudes or aspirations. The cross-country variation is, moreover, strongly patterned by broad cultural regions (e.g. smaller in East and Southeast Asia, larger in the Nordics and Middle East/Central Asia), pointing to the role of broader gender norms.

Gender norms are often viewed as detrimental to girls; our findings suggest that they can also be harmful to boys in terms of educational outcomes—academic performance, continuation into tertiary education, and EYS. This is notably the case for a gendered conception of ability that associates talent and brilliance more with boys and effort and diligence more with girls (23–26, 47). These two facets may work together: girls may doubt their innate talent and compensate through effort—effort that is itself culturally coded as feminine—while boys may rely on a presumed talent and devalue an effort coded as feminine. Such a gendered conception, usually seen as detrimental to girls, may thus also disadvantage boys in their relation to academic effort. It is, however, worth noting that although girls’ more positive attitudes toward academic effort are associated with higher access to tertiary education, this does not necessarily translate into greater representation in the most prestigious or lucrative fields, such as STEM. More generally, boys’ lower academic achievement does not necessarily translate into worse outcomes in the labor market (50).

Addressing differences in attitudes may be a key to advancing educational equity for both genders. The main policy implication emerging from our findings is the need to foster more positive attitudes toward academic effort among boys, particularly in economically developed countries. Our analysis suggests that promoting a growth mindset (45, 46, 51) and emphasizing that success for both girls and boys is achieved through learning and effort could help address the “boys’ crisis” and foster greater gender equality in education. Gender norms, which often discourage boys from engaging academically due to traditional expectations of masculinity, must be challenged. Interventions should specifically target encouraging boys to participate in fields where they are underrepresented, such as the humanities, and role models who defy traditional gender roles—both in terms of attitudes toward academic effort and areas of study—could inspire boys, challenge stereotypes, and contribute to a more inclusive education system.

### Our analysis has several limitations

First, we measure students’ attitudes toward academic effort rather than effort itself; these attitudes are self-reported and may be subject to biases such as social desirability. Nonetheless, attitudes toward effort are of intrinsic interest, and we show that our measures are significantly associated with PISA scores for both boys and girls at the individual level. Our primary measure (THI) is, moreover, a single item, precluding formal cross-national invariance testing; this is partly mitigated by our focus on within-country gender gaps rather than absolute cross-national comparisons and by the full replication of results using the multi-item ATTLNACT index (see Supplementary material). Second, our analysis focuses on 15-year olds, an age at which stereotypes may strongly influence academic attitudes, so our findings may not generalize to other ages.

Third, the analysis is correlational rather than causal: although we suggest that gender norms and gender differences in ability beliefs shape attitudes, which in turn influence achievement and choices, bidirectional relationships are likely—for instance, boys’ underrepresentation in tertiary education may itself lead them to devalue school, reinforcing performance gaps.

Finally, our analysis is limited to countries participating in PISA and may not generalize to contexts where girls’ access to schooling remains severely restricted. It also does not examine how institutional features of education systems—such as the relative weight of standardized examinations in admissions—might shape gender differences in effort-related attitudes. Future research using longitudinal or quasi-experimental designs, alternative datasets, and measures of effort and norms would help clarify these mechanisms.

## Materials and methods

### Data

We draw on PISA, the OECD's triennial assessment of 15-year olds in mathematics, reading, and science. Our primary data come from the 2012 and 2018 waves, the only two that include the THI and ATTLNACT measures. We use these data to measure students’ attitudes toward academic effort, academic achievement, and individual covariates and to construct several country-level gender gaps (e.g. in growth mindset, fear of lacking talent, and parental support). We complement PISA with country-level indicators of socioeconomic development and gender equality, measures of gender disparities in education, and measures of gender stereotypes from external sources. See Appendices A and B for documentation of the PISA surveys, variable definitions, item codes, and all data sources.

### Methods

PISA uses a two-stage stratified probability design: schools are sampled within each country (stratified by region/type/size), and then 15-y-old students are randomly selected within sampled schools. We analyze PISA 2012 and PISA 2018 microdata, following OECD guidance for inference under this complex survey design. Achievement is treated via plausible values as multiple imputations. All estimates use student weights with balanced repeated replication/Fay replicate weights to obtain design-consistent SEs. We apply “senate” weighting—normalizing student weights to sum to one within each country–year, with the same scaling factor applied to all 80 replicates, ensuring equal contribution of each country–year observation. Unless otherwise noted, variables are standardized within country–year (mean 0, SD 1), so coefficients are expressed in within-country–year SD units. The average gender gap in effort-related attitudes is estimated on the pooled sample with country and year fixed effects; the coefficient is thus interpreted as the average G - B difference in within-country–year SD units, holding country and year constant. Analogous models produce country-specific and country-by-year gaps. Cross-country associations are estimated by OLS on standardized variables with heteroskedasticity-robust SEs. Spatial diagnostics (Moran's *I*) and spatial error models (GS2SLS) are reported in the Supplementary material and confirm robustness to spatial dependence.

Further details, including full model specifications, are provided in Appendix C.

## Supplementary Material

pgag316_Supplementary_Data

## Data Availability

A replication package containing the analysis datasets and code is available on the Open Science Framework at https://doi.org/10.17605/OSF.IO/VDMYK. The underlying survey data come from the OECD Programme for International Student Assessment 2012 and 2018 and are publicly available at https://www.oecd.org/pisa/data/.
