# Supplementary material for: Gender differences in attitudes toward academic effort and boys’ underachievement: A large-scale international study
Source: PNAS Nexus. 2026 Oct 13;5(10):pgag316. doi: 10.1093/pnasnexus/pgag316 (PMC13659772; doi:10.1093/pnasnexus/pgag316)
Supplement: Supplementary file 1 [file pgag316_supplementary_data.pdf]

Supplementary Information for
Gender Differences in Attitudes towards Academic Effort and Boys' Underachievement: a Large-Scale International Study

Author: Clotilde Napp[a,b,*]

Affiliations:
[a] CNRS, UMR7088, 75016 Paris, France
[b] Université Paris-Dauphine, PSL Research University, Paris, France

[*]Corresponding author: Clotilde Napp, Université Paris-Dauphine, Place du Maréchal de Lattre de Tassigny, 75016 Paris, France.

Email: clotilde.napp@dauphine.psl.eu

This PDF file includes:

Supplementary text: Appendix A-E
Figures S1 to S4
Tables S1 to S3
SI References

Gender Differences in Attitudes towards Academic Effort and Boys' Underachievement: a Large-Scale International Study 1
Appendix A: PISA 2
General Description 2
Data on attitudes towards academic effort 3
Data on academic performance 4
Data for individual controls and subgroups of students 4
Appendix B: Other Data 5
Country-level measures of development, gender equality and individualism 5
Country-level measures of gender disparities in education 7
Country-level measures of gender differences in growth mindset/parental support and gender norms 9
Appendix C: Methods 10
PISA design and estimation 10
i. PISA sampling, plausible values and inference 10
ii. Weights and representativeness 11
iii. Standardized variables 11
Individual-level models 11
Individual-level models: Estimating gender gaps 11
Individual-level models: Testing cross-national variation 12

Other individual-level models 13
Country-level models 15
Associations between gender gaps in attitudes towards effort and development indicators and gender norms 15
Associations between gender gaps in attitudes towards effort and gender gaps in educational outcomes 15
Appendix D: Main Figures and Tables 17
Figures 17
Tables 21
Appendix E: Robustness to Spatial Dependence: GS2SLS spatial error models 40
Global Moran's I on the dependent variable 41
Table S1D using spatial error models 41
Table S2B using spatial error models 43
Table S2C using spatial error models 44
Table S2D using spatial error models 45
Table S3B using spatial error models 47

Supplementary Information Text
Appendix A: PISA

General Description

The Programme for International Student Assessment (PISA) is an every-three-year international survey of 15-year-old students aimed at determining their knowledge and skills in different domains. Students' abilities are assessed in the three curricular domains: mathematics, reading, and science. Students also answer a background questionnaire, seeking information about the students themselves, their homes, and their school and learning experiences.
The PISA target population is made up of all students in any educational institution between the ages of 15 years and 3 months and 16 years and 2 months at the time of the assessment. This specific age has been chosen because it is close to the end of compulsory education in most countries. Efforts have been made to ensure the absence of cultural or national biases in the test items and in the evaluation of performance.

On top of taking math, reading and science tests, students fill out a background questionnaire that provides contextual information about themselves, their homes, and their school and learning experiences. The background questionnaire takes about 30 minutes to complete. It includes general questions about gender, home possessions, parental occupation and education and also seeks more specific information about students' engagement with and at school. Of particular interest to us are questions about their attitudes towards academic effort (see details below).

We analyse data from the PISA 2012 and the PISA 2018 surveys. We focus on these two surveys because they are the only ones including questions about attitudes towards academic effort. The student data set in 2012 contains around 450,000 observations, which roughly represent a population of 28 million 15-year-olds attending seventh grade or above in 64 countries, including the 34 countries belonging to the Organisation for Economic Co-operation and Development (OECD) in 2012. The student data set in 2018 contains around 600,000 observations, representing a

population of roughly 32 million 15-year-olds attending seventh grade or above in 79 countries, 37 of which belong to the OECD in 2018, covering in total students from 80% of the world economy.

PISA surveys systematically assess students' performance and knowledge in three core subjects: mathematics, reading and science. However, one of the three core subjects is chosen to be covered in greater depth in each survey. In 2012, mathematics literacy is the major subject area, (as it was in 2003) and in 2018, reading literacy is the major subject area (as it was in 2000 and 2009).

Data description. We merge data from PISA2012 and PISA2018. We only keep observations that are representative of countries that are included in both surveys. This leaves us with 966,044 observations in our sample, from 61 countries, with a minimum (resp. maximum) number of observations per country of 6,804 for Iceland (resp. of 61,256 for Spain). Some items (but not those about attitudes towards academic effort) are included in only one of the two surveys.

Data on attitudes towards academic effort

Attitudes towards the importance of trying hard at school (*THI*). In the background questionnaire, PISA2012 and PISA2018 ask students, *thinking about their school, how much they agree with the following statement 'Trying hard at school is important'* (PISA 2012: ST91Q03; PISA 2018: ST182Q03HA) (1. "Strongly agree", 2. "Agree", 3. "Disagree", 4. "Strongly disagree"). We consider the opposite of the variable so that higher values correspond to more positive attitudes towards trying hard at school, and unless otherwise specified, the variable is standardized within country-year. As a single-item measure, THI does not permit formal multi-group invariance testing. This limitation is mitigated by three factors: our primary estimands are within-country gender gaps rather than cross-country absolute comparisons; scores are standardized within country-year; and results replicate identically using the multi-item ATTLNACT index, for which PISA documents rigorous cross-national scaling and high internal consistency (median Cronbach's α = 0.80 across OECD countries in PISA 2012). Our sample contains 764,461 observations from 61 countries.

The index of Attitudes towards learning activities (*ATTLNACT*) was constructed by PISA2012 and PISA2018 based on a trend question asking students whether they agree ("strongly disagree", "disagree", "agree", "strongly agree") with the following school-related statements: "*Trying hard at school will help me get a good job* "; "*Trying hard at school will help me get into a good college*"; "*Trying hard at school is important*"; and "*I enjoy getting good grades*" (PISA 2012: ST91Q01- ST91Q04; PISA 2018: ST182Q01HA-ST182Q04HA). This index is used as provided by PISA without modification. Positive values on this scale mean that the student has more positive attitudes than the average student across OECD countries towards learning activities and a higher value of school. The OECD reports high internal consistency for ATTLNACT across participating countries. In PISA 2012, the median Cronbach's alpha for ATTLNACT was 0.80 across OECD countries and 0.79 across partner countries/economies (PISA 2012 Technical Report, Tables 16.39 and 16.40). PISA 2018 documentation likewise reports high internal consistency for this index across countries (see PISA 2018 Technical Report, Tables 16.29 and 16.30 or the corresponding OECD internal-consistency tables). Unless otherwise specified, the

variable is standardized within country-year. Our sample contains 762,935 observations from 61 countries.

Data on academic performance

Academic performance in math, reading and science and general performance. We use individual-level PISA scores in math, reading and science to measure students' performance. These scores are on a 0-1000 scale. They have been scaled during the first PISA survey in 2000 to have a mean of 500 and a standard deviation of 100. Unless otherwise specified, we consider the score standardized within country-year. We also consider general performance, measured as an equally weighted average of the three (raw) scores in math, reading and science. Unless otherwise specified, the variable is standardized within country-year. There are 960,667 observations from 61 countries in our sample with information about performance in math and science, and 924,724 observations from 61 countries with information about performance in reading.

Low performers. We consider the likelihood of being a low performer in reading and a low performer in general. We define low performers as students performing below their country's mean in the given PISA cyle, i.e., with negative standardized (within country-year) scores.

Data for individual controls and subgroups of students

Individual characteristics include socioeconomic background, the grade attended by the student, grade repetition, the age of the student, the level of education of the student's parents, measured both in years and kind of diploma obtained, a measure of home educational resources, the family structure (parents at home or not), measures of truancy and a measure of how much students think that they belong to school.

- **Socioeconomic background.** The PISA index of Economic, Social and Cultural Status (ESCS) is derived from three variables related to family background: parents' highest level of education (PARED), parents' highest occupational status (HISEI), and home possessions (HOMEPOS), including books in the home. When considering student subgroups, we define a student with low socioeconomic background as a student with negative standardized level of ESCS, i.e., as a student whose level of socioeconomic background ESCS is below the average in the student's country. There are 941,611 observations from 61 countries in our sample with information on ESCS.
- PISA provides indices on **parental education**. Indices with these categories were provided for the student's mother (MISCED) and the student's father (FISCED). PISA also provides the index of highest education level of parents (HISCED) corresponding to the higher ISCED level of either parent, also recoded into estimated number of years of schooling (PARED). When considering student subgroups, we refer to a high level of education for ISCED level 6 (bachelor's or higher level corresponding to more than 15 years of education) and a low level of education for ISCED level 0 or 1 (primary education corresponding to less than 6 years of education). There are between 906,082 and 938,144 observations from 61 countries in our sample with information on parental education.
- PISA provides an index measuring **home educational resources** (HEDRES). There are 945,142 observations from 61 countries in our sample with information on HEDRES.
- PISA includes questions about **grade repetition**. The grade repetition variable (REPEAT) is a dummy equal to one if the student has repeated a grade in at least one ISCED level. There are 908,503 observations from 61 countries in our sample about grade repetition.

- PISA provides information about the **grade** attended by the student. The *modal* grade is defined as the level attended by at least one-third of the PISA sample. When considering student subgroups, a low (resp. high) grade is a grade lower (resp. higher) than the modal grade. There are 963,934 observations from 61 countries in our sample about grade repetition.
- PISA provides information about the **age** of the student. There are 965,928 observations from 61 countries in our sample about student age.
- PISA2012 (but not PISA2018) provides information about the **family structure** of the student and in particular if the father is absent or present at home (st11q02) and if the mother is absent or present at home (st11q01). There are 425,316 observations from 61 countries in our sample with information about family structure.
- PISA includes questions about students' **truancy**, and in particular about *arriving late at school* (PISA 2012: ST09Q01; PISA 2018: ST062Q01TA), *skipping classes* (PISA 2012: ST115Q02; PISA 2018: ST062Q03TA), or *skipping days of school* (PISA 2012: ST115Q01; PISA 2018: ST062Q02TA) *in the two weeks prior to the PISA test*. We also introduce a measure of truancy by considering the weighted average of the three (standardized within country-year) variables of truancy, and re-standardized within country-year. There are 872,396 observations from 61 countries in our sample with information about truancy. When considering student subgroups, we define a student with low level of truancy as a student with negative standardized (within country-year) level of truancy, i.e., as a student whose level of truancy is below the average in the student's country in the given PISA cycle.
- The index of **sense of belonging** (BELONG) was constructed using students' responses to a trend question about their sense of belonging at school (PISA 2012: ST87Q01–ST87Q06; PISA 2018: ST034Q01TA–ST034Q06TA). Students were asked whether they agree ("strongly disagree", "disagree", "agree", "strongly agree") with the following school-related statements: "*I feel like an outsider (or left out of things) at school*"; "*I make friends easily at school*"; "*I feel like I belong at school*"; "*I feel awkward and out of place in my school*"; "*Other students seem to like me*"; and "*I feel lonely at school*". Items reflecting lack of belonging ("I feel like an outsider", "I feel awkward and out of place in my school", "I feel lonely at school") are reverse-coded by PISA so that higher values consistently correspond to a greater sense of belonging. This index is used as provided by PISA without modification. Positive values in this scale correspond to a greater sense of belonging at school than the average student across OECD countries. There are 747,479 observations from 61 countries in our sample with information about sense of belonging.

Appendix B: Other Data

Our primary data source is the Programme for International Student Assessment (PISA2012 and PISA2018). We complement these data with a range of country-level indicators including measures of country socioeconomic development and gender equality, measures of gender disparities in education, and measures of gender differences in attitudes (such as growth mindset) and gender norms.

Country-level measures of development, gender equality and individualism

PISA Index of economic, social and cultural status (ESCS)

The PISA index of Economic, Social and Cultural Status (ESCS) is derived from three variables related to family background: parents' highest level of education, parents' highest occupational status and home possessions (see Appendix A). We consider the mean value by country of the average of the PISA2012 and PISA2018 indices. Data are available for 61/61 countries in our sample.

Source: PISA2012 and PISA2018

Human Development Index (HDI)

The Human Development Index (HDI) is a composite statistic of life expectancy, education, and per capita income indicators. A country scores a higher HDI when life expectancy, education level and per capita income is higher. We consider the average of the values for year 2012 and 2018. We also consider an historical value of HDI with the value in 1990. Data for the main HDI value (resp. HDI1990) are available for 59/61 (resp. 52/61) countries in our sample.
Source: Human development report 2013, Human development report 2019, UNDP
https://hdr.undp.org/system/files/documents/global-report-document/hdr2013encomplete.pdf

Gross Domestic Product (GDP)
GDP per capita is gross domestic product divided by midyear population. GDP is the sum of gross value added by all resident producers in the economy plus any product taxes and minus any subsidies not included in the value of the products. It is calculated without making deductions for depreciation of fabricated assets or for depletion and degradation of natural resources.
Our main measure of GDP is the value for 2012. Data are in constant 2017 international dollars and are available for 60/61 countries in our main sample.
We also consider historical values of GDP (GDP 1960, GDP 1970, GDP 1980), Data are in constant 2015 US dollars and are available for 40/61 countries for GDP1980, 34/6**1** countries for GDP1970 and 31/61 countries for GDP1960.
We consider the log of the GDP values.
Source:
Main value: GDP2012 per capita PPP cstt 20107 international $, worldbank
https://data.worldbank.org/indicator/NY.GDP.PCAP.PP.KD
Historical values: GDP1960-1970-1980 Per capita cstt 2015US$, worldbank
https://data.worldbank.org/indicator/NY.GDP.PCAP.KD

Gender Gap Index (GGI)
The Gender Gap Index, from the World Economic Forum, synthesizes the position of women in any given country by taking into account economic opportunities, economic participation, educational attainment, political achievements, and health and well-being. Larger values point to a better position of women in society. Values have been taken for 2012 and for 2018 and we consider the average of these values. The GGI measure is available for 56/61 countries in our sample.
Source: GGI2012, World economic forum
https://www3.weforum.org/docs/WEF_GenderGap_Report_2012.pdf
GGI2018, World economic forum

Individualism (Hofstede)
Our variable of individualism is based on Hofstede's cultural dimensions (1). It captures the degree to which a society is individualistic as opposed to collectivist, *i.e.,* the extent to which individuals are integrated into groups, and how loose are social links. The individualism measure is available for 53/61 countries in our main sample.
Source: https://www.hofstede-insights.com/product/compare-countries/

Parasite-Stress
We use the data gathered by (2). The combined parasite stress measure is a measure of infectious diseases and combines a measure of morbidity and mortality DALY

attributed to 28 different infectious and parasitic diseases for the year 2002, *e.g.,* tuberculosis, measles, leprosy or dengue (Disability Adjusted Life Years; DALY) and the Prevalence of 'nonzoonotic' (i.e., human-specific and multi-host infectious diseases) infectious and parasitic diseases. The DALY measure combines the time lived with disability and the time lost due to premature mortality. Data are available for 57/61 countries in our main sample.
Source: (2), Electronic Supplement ES2
http://www.journals.cambridge.org/bbs2012002
https://www.cambridge.org/core/journals/behavioral-and-brain-sciences/article/abs/parasitestress-promotes-ingroup-assortative-sociality-the-cases-of-strong-family-ties-and-heightened-religiosity/0331C3331E16F6C15BB9A5AF1AA07108#supplementary-materials

Country-level measures of gender disparities in education

Gender differences in enrolment in tertiary education. We rely on OECD data about enrolment in tertiary education by gender and by field of study. We consider by country the number of males and the number of females, aged 19-22, who are enrolled in tertiary education in 2017. We then take the ratio of the number of males over the sum of the number of males and females as the share of enrolled students in tertiary education who are males. Data are available for 40/61 countries in our sample.
Since Israel is specific because males of this age are enrolled in the army, we provide results with and without Israel.
Source: https://stats.oecd.org/# Data extracted on 19 Mar 2024 09:02 UTC (GMT) from OECD.Stat

Gender differences in expected years of schooling. Expected Years of Schooling (EYS) is the number of years a child of school entrance age is expected to spend in the education system (at school or university, including years spent on repetition). It is the sum of the age-specific enrolment ratios for primary, secondary, post-secondary non-tertiary and tertiary education.
School life expectancy indicates the average number of years of schooling that the education system can offer. A high value indicates a probability for children to spend more years in education
Expected years of Schooling is used to compare educational access across countries and between genders. As underlined by Unesco, a significant gap between boys and girls may indicate gender disparities in education. Data (for the most recent year available) are available for 59/61 countries in our sample.
Source:
UNESCO Institute for Statistics (UIS). UIS.Stat Bulk Data Download Service. Accessed September 30, 2024. https://apiportal.uis.unesco.org/bdds.
Source: https://genderdata.worldbank.org/indicators/se-sch-life/

Gender differences in enrolment in tertiary Arts and Humanities. We rely on OECD data about enrolment in tertiary education by gender and by field of study (see above). We consider by country the number of males and the number of females, aged 19-22, who are enrolled in tertiary education in the field of Arts and Humanities in 2017. We then take the ratio of the number of males enrolled in this field over the number of

males and females as the (absolute) share of students in the field of Arts and Humanities who are males. A share of 50% would correspond to an equal representation. Data are available for 40/61 countries in our sample.
To capture the underrepresentation of males in this specific field of study relative to the other fields, we consider the (relative) share of students in the field of Arts and Humanities who are males, measured by the ratio of the absolute share of males in Arts and Humanities over the share of males in all fields of tertiary education.
Source: https://stats.oecd.org/#

Gender differences in enrolment in tertiary STEM (Science, Technology, Engineering and Mathematics). We rely on OECD data about enrolment in tertiary education by gender and by field of study (see above). We consider by country the number of males and the number of females, aged 19-22, who are enrolled in tertiary education in the STEM fields in 2017. We then take the ratio of the number of males enrolled in this field over the number of males and females as the (absolute) share of students in the field of Arts and Humanities who are males. A share of 50% would correspond to an equal representation. Data are available for 40/61 countries in our sample.
To capture the underrepresentation of males in this specific field of study relative to the other fields, we consider the (relative) share of students in the STEM fields who are males, measured by the ratio of the absolute share of males in STEM over the share of males in all fields of tertiary education.
Source: https://stats.oecd.org/#

Gender differences in Intentions to pursue math studies and careers. Data are from PISA2012. PISA2012 asks students to report their intentions to use mathematics in their future studies and careers. Five items (ST48) use the forced-choice format to measure students' plans regarding mathematics at some stage in the future. The first item has students decide between taking additional courses in mathematics or the language of the test after school finishes. The second item asks whether students plan on majoring in a subject requiring mathematics or science at college (or equivalent educational institution in different countries). The third item asks whether students are willing to study harder in either their mathematics classes or in classes teaching the language of the test. For the fourth item, respondents have to indicate whether they are planning on taking as many mathematics or science classes as possible during their education. The fifth and last item of that battery requires respondents to choose whether they are planning on pursuing a career that involves a lot of mathematics or science. PISA2012 uses students' responses to these five items to create the index (MATINTFC), reflecting the extent to which a student intends to pursue math studies and careers. All items are reversed so that respondents who choose mathematics over either science or the test language are allocated a higher code. To compute country gender gaps in intentions to pursue math studies and careers, we proceed as in Model (1) below (Appendix C). Data are available for 61/61 countries in our sample.
Source: PISA2012

Gender differences in expectations of a career in health. Data are from PISA2018. PISA2018 asks students what occupation they expect to be working in by the time they are 30 years old. Responses to this open-ended question are re-classified according to the International Standard Classification of Occupations 88 (ISCO88;

International Labor Office, 1988). For each country, we consider the difference between the percent of all 15y.o. females and the percent of all 15y.o. males participating in the survey who expect a career in health, which indicates the extent to which females expect more than males a career in health in the country. Data are available for 61 countries in our sample. These data deal with expectations and not real choices of occupations.
Source: PISA2018 Results, vol.II: Where all students can succeed. Table II.B1.8.19

Country-level measures of gender differences in ability beliefs (growth mindset/fear of lacking talent) and parental support and of gender stereotypes

Gender differences in growth mindset. Data are from PISA2018. PISA2018 asks students whether they agree ("strongly disagree", "disagree", "agree", "strongly agree") with the following statement (ST184Q01HA): "*Your intelligence is something about you that you can't change very much*". Students who disagree with the statement are considered to have a growth mindset. For each country, we consider the difference between the percentage of girls and the percentage of boys (G-B) who disagreed or strongly disagreed with the statement. Data are available for 60/61 countries in our sample.
Source: PISA2018 Results, vol.III: Where all students can succeed. Table III.B1.14.3

Gender differences in Attribution of failure to lack of talent. Data are from PISA2018. Students in PISA 2018 were asked to report the extent to which they agree ("strongly disagree", "disagree", "agree", "strongly agree") with the following statements (ST183q02): "*When I am failing, I am afraid that I might not have enough talent*". Students who agree with the statement are considered to attribute failure to lack of talent. For each country, we consider the difference between the percentage of girls and the percentage of boys (G-B) who agreed or strongly agreed with the statement. Data are available for 59/61 countries in our sample.
Source: PISA2018 Results, vol.II: Where all students can succeed. Table II.B1.8.18

Gender differences in perceived parental support. Data are from PISA2018. Students in PISA 2018 were asked to report the extent to which they agree ("strongly disagree", "disagree", "agree", "strongly agree") with the following statement: *"Thinking about this academic year, my parents support my educational efforts and achievements" (*ST123Q02NA). For each country, we consider the difference between the percentage of girls and the percentage of boys (G-B) who strongly agreed with the statement. Data are available for 59/61 countries in our sample.
Source: PISA2018

Implicit gender stereotypes about career and family. Our measures of gender career–family stereotypes rely on data from Project Implicit (3) (http://implicit.harvard.edi/implicit/) collected during the years 2005–2020. In Project Implicit, the gender career implicit association test (IAT), introduced in the literature in (4), permits to detect the implicit associations between career and men and between family and women. The relative speed at which the subject can pair categories (men and women) and attributes (career and family) measures implicitly the strength of the

association between these categories and attributes. We rely on (5) for data about country level measures of implicit stereotypes. Data are available for 53/61 countries in our sample.
Source: (5)

Gender communality and agency stereotypes. Our measures of gender communality and agency stereotypes rely on data collected between 2019 and 2020 as part of a large cross-cultural project Towards Gender Harmony (6). For a set of traits related to communion (compassionate, helpful to others, sympathetic, understanding of others, aware of others' feelings, devoted to others, warm, supportive) and to agency (competent, confident, has leadership abilities, determined, courageous, active, capable, independent), participants rated "*How desirable is it in your society for a woman [man] to possess this trait?*" on a scale of 1 (not at all desirable) to 7 (very desirable). Our measure of gender communality stereotypes is the country-level average, across all communion-related traits, of the difference between the desirability rating for women and that for men (W − M); a higher value thus indicates that communal traits are more strongly prescribed for women than for men. Our measure of gender agency stereotypes is constructed analogously across all agency-related traits, but as the difference between the desirability rating for men and that for women (M − W); a higher value thus indicates that agentic traits are more strongly prescribed for men than for women. In both cases, a higher value corresponds to a stronger traditional gender stereotype. Data are available for 42/61 countries in our sample.
Source: (7)

Appendix C: Methods

PISA design and estimation

i. PISA sampling, plausible values and inference

PISA uses a two-stage stratified probability design: schools are sampled within each country (stratified by region/type/size), then 15-year-old students are randomly selected within sampled schools (grade-independent). All analyses use student weights to correct for unequal selection and nonresponse, and replicate weights (Balanced Repeated Replication with Fay's adjustment) to obtain valid standard errors under the complex design.
Achievement in math, reading, and science is reported as plausible values (PVs)—multiple draws from each student's posterior ability distribution under Item Response Theory (5 PVs in PISA 2012; 10 PVs in PISA 2018). Following OECD practice, we treat PVs as multiple imputations: for any statistic (e.g., a gender gap or regression coefficient), we (i) compute the estimate within each PV using the full set of survey and replicate weights, and (ii) take the arithmetic mean across PVs as the point estimate. Standard errors combine (a) sampling variance estimated via the 80 BRR replicate weights, and (b) imputation variance across PVs, so total uncertainty reflects both the sample design and proficiency measurement error.
All individual-level results in this paper (and in particular the computation of gender gaps in effort-related attitudes or in achievement) follow this procedure. In practice,

we implement it in Stata with the repest module (OECD, (8)), which automates BRR/Fay replicate weighting for PISA and related large-scale assessments.

ii. Weights and representativeness

PISA supplies student weights so that each country's sample is representative of its population of 15-year-olds. We use these weights in all country-level and pooled analyses to obtain design-consistent estimates that are not distorted by unequal selection or nonresponse. In the pooled file, the sum of original PISA weights is proportional to each country's population, so more populous countries weigh more in world-level statistics. Because our target is cross-country inference with the country as the unit of analysis, we instead use "senate" weights—i.e., we normalize student weights to sum to one within each country-year (country x survey cycle) on the analysis sample—so that each country contributes equally in each cycle regardless of population size. This convention aligns with the way PISA reports country statistics (e.g., national gender gaps) and prevents trends in a few very populous countries from dominating average cross-country relationships. Note that the same country-year-specific scaling factor is applied to all 80 BRR replicate weights, preserving the replicate structure and yielding design-consistent standard errors.

When combining PISA 2012 and PISA 2018, senate weighting has an additional benefit: it gives equal footing to each cycle. Because the original student weights are cycle-specific, normalizing within country-year ensures that countries that appear in both surveys contribute equally by country and by cycle, rather than being driven by cycle-specific weighting differences.

iii. Standardized variables

Unless otherwise noted (e.g., for indicator variables), all variables derived from PISA are standardized within country- year to have mean 0 and standard deviation 1. This country-year standardization (i) ensures scale comparability across countries and survey cycles and (ii) makes gender gaps directly interpretable in standard-deviation units. In particular, our measures of attitudes toward academic effort are standardized within country-year before computing country-specific and pooled estimates. Hence, coefficients in subsequent models— and in particular the estimates of gender gaps—are expressed in within–country–year SD units.

Individual-level models

Unless otherwise specified, individual-level models use senate weights (student weights normalized to sum to one within country-year) and BRR/Fay replicate weights, yielding design-consistent standard errors that account for PISA's complex survey structure. Attitudes toward academic effort (THI or ATTLNACT) are standardized within country-year so that coefficients are expressed in within-country-year SD units. Deviations from this procedure (e.g., hierarchical models, unweighted models) are explicitly noted below.

Individual-level models: Estimating gender gaps

i. Average gender gap (pooled sample)

Model (1a): $Y_{ict} = \alpha_c + \delta_t + \beta \, Girl_{ict} + \varepsilon_{ict}$

where Y_{ict} is attitudes toward academic effort (THI or ATTLNACT), standardized within country-year; $Girl_{ict}$ is a dummy variable equal to 1 if student i is a girl; α_c are country fixed effects; δ_t are year fixed effects; and ε_{ict} is the error term. The

coefficient β captures the average within-country gender gap across countries and years (Table 1, Table S1A Panel A).

ii. Country-specific gender gaps
For each country c, we estimate:
Model (1b): $Y\_ict = \gamma\_c + \delta\_t + \beta\_c \text{ Girl\_ict} + \varepsilon\_ict$
where γ_c is the intercept for country c, and δ_t are year fixed effects pooling 2012 and 2018 within country c. The coefficient β_c is the gender gap for country c in SD units (Table S1A Panel B). These country-level gaps are used as outcomes in cross-country regressions (Section below).

iii. Country-by-year gender gaps
For descriptive purposes and sensitivity checks, we also estimate gaps separately for each country-year combination (c,t):
Model (1c): $Y\_ict = \gamma\_\{ct\} + \beta\_\{ct\} \text{ Girl\_ict} + \varepsilon\_ict$
where $\gamma\_\{ct\}$ is the intercept for country c and year t. The coefficient $\beta\_\{ct\}$ is the gender gap for country c in year t in SD units (Table S1A Panel B reports both pooled and year-specific estimates).

iv. Gender gaps with individual controls
Model (1d): $Y\_ict = \alpha\_c + \delta\_t + \beta \text{ Girl\_ict} + \gamma' X\_ict + \theta' (\text{Girl\_ict} \times X\_ict) + \varepsilon\_ict$
where X_ict includes ESCS, parental education, grade repetition, home resources, and sense of belonging (all standardized within country-year). The coefficient β is the adjusted gender gap at the mean of the standardized covariates (Table S1B).

v. Gender gaps in subsamples
We also estimate model (1a) on specific subsamples (e.g., high/low ESCS, repeaters/non-repeaters), with weights renormalized within each subsample (Table 1, Table S1C).

Individual-level models: Testing cross-national variation

To test whether gender gaps vary systematically with country-level socio-economic development, we estimate individual-level interaction models that address methodological concerns raised recently in the literature (e.g., (1–3)) regarding the interpretation of cross-national difference scores.

Individual-level interaction model

Model (2): $Y\_ict = \alpha\_c + \delta\_t + \beta_1 \text{ Girl\_ict} + \beta_2 (\text{Girl\_ict} \times ESCS\_c) + \varepsilon\_ict$

where Y_ict is attitudes toward academic effort (THI or ATTLNACT), standardized within country-year; Girl_ict is a dummy variable equal to 1 if student i is a girl; ESCS_c is the country-level mean of the PISA Economic, Social and Cultural Status index, standardized across countries; α_c are country fixed effects; δ_t are year fixed effects; and ε_ict is the error term. The coefficient β_2 captures how the within-country gender gap (in SD units) varies with country-level socioeconomic development.

This specification directly tests whether the within-country gender gap varies systematically with country-level development, providing an individual-level analogue to regressions of aggregated gender gaps on country characteristics and reducing reliance on a two-step difference-score approach.

Robustness specifications

We estimate six variants of model (2) to assess robustness across measurement and modeling choices:

(i) Design-consistent PISA estimation (primary specification): Model (2) is estimated using senate weights and BRR/Fay replicate weights following the procedures described above.

(ii) Cluster-robust standard errors: Model (2) re-estimated using analytical weights with standard errors clustered at the country level, providing inference robust to within-country correlation of observations.

(iii) Hierarchical linear model: A random-intercept multilevel model with country-specific intercepts ($u_0c \sim N(0, \tau^2)$), and fixed effects for gender, country-level ESCS, their interaction and year. Estimated via maximum likelihood with probability weights and robust standard errors.

(iv) Non-standardized outcome / gender-specific slopes: Model (2) re-estimated using raw (non-standardized) Y_ict to rule out artifacts of within-country-year standardization, without country fixed effects, and including both country-level ESCS and its interaction with gender, so that ESCS slopes can be identified separately for boys and girls.

(v) Unweighted estimation: Model (2) estimated without survey weights, using cluster-robust standard errors.

(vi) Individual-level controls: Model (2) augmented with individual covariates X_ict (grade repetition, parental education, student-level ESCS, home educational resources, sense of belonging at school), all standardized within country-year.

Results from all six specifications are reported in Table S1E.

Other individual-level models

a. Association between attitudes towards academic effort and academic performance

We estimate:

Model (3a):

$$\text{Performance}\_{ict} = \alpha\_c + \delta\_t + \beta\, Y\_{ict} + \varepsilon\_{ict}$$

where Performance_ict is student i's performance in math, reading, science, or an aggregate score (standardized to mean 0, SD 1 within country-year); Y_ict is attitudes toward academic effort (THI or ATTLNACT), standardized within country-year; α_c are country fixed effects; and δ_t are year fixed effects. Estimation follows the same weighting and variance procedures as models (1a)-(1e): senate weights and BRR/Fay replicate weights. For performance measures, plausible values are combined following OECD guidance.

The coefficient β measures the association between effort attitudes and performance (both in SD units), holding country and year constant.

We estimate this model for all students, as well as separately for boys and girls, to obtain gender-specific association coefficients.

To test whether the association between effort attitudes and performance differs significantly by gender, we use an interaction specification:

Model (3a'):

$$Performance\_ict = \alpha\_c + \delta\_t + \beta_1\ Girl\_ict + \beta_2\ Y\_ict + \beta_3\ (Girl\_ict \times Y\_ict) + \varepsilon\_ict$$

where Girl_ict is a dummy variable equal to 1 if student i is a girl. The coefficient β_3 captures the difference between girls' and boys' effort-performance slopes. A significant β_3 indicates that the association between attitudes and performance differs by gender.

Results are reported in Table S2A. We additionally examine the likelihood of being a low performer (performing below the country-year median) in overall performance and in reading specifically, using logistic regression with the same fixed effects structure (Tables S2A).

b. Association between growth mindset and attitudes towards effort

To test whether students' beliefs about intelligence malleability predict their valuation of academic effort, we estimate:

Model (3b):

$$Y\_ict = \alpha\_c + \delta\_t + \beta\ GrowthMindset\_ict + \varepsilon\_ict$$

where Y_ict is attitudes toward academic effort (THI or ATTLNACT), standardized within country-year; GrowthMindset_ict is student i's growth mindset (reverse-coded disagreement with "Your intelligence is something you can't change very much"), standardized within country-year; α_c are country fixed effects; and δ_t are year fixed effects. Estimation follows senate weights and BRR/Fay replicate weights.

The coefficient β measures the association between growth mindset and effort attitudes (both in SD units), holding country and year constant.

We also estimate this model separately by gender (Table S3A). To test whether the association between effort attitudes and growth mindset differs significantly by gender, we use an interaction specification:

Model (3b'):

$Y\_ict = \alpha\_c + \delta\_t + \beta_1$ Girl_ict + β_2 GrowthMindset_ict + β_3 (Girl_ict × GrowthMindset_ict) + ε_ict

where Girl_ict is a dummy variable equal to 1 if student i is a girl. The coefficient β_3 captures the difference between girls' and boys' effort-growth mindset slopes. A significant β_3 indicates that the association between attitudes and growth mindset differs by gender.

Country-level models

We study cross-country associations using OLS regressions with heteroskedasticity-robust standard errors. All variables are standardized within the regression sample (mean 0, SD 1), so coefficients represent the change in SD units of the dependent variable associated with a 1-SD increase in the explanatory variable. Standardized OLS coefficients are algebraically equivalent to Pearson correlation coefficients.

Associations between gender gaps in attitudes towards effort and development indicators and gender norms

Model (4a): gapEffort_c = $\beta_0 + \beta_1$ Z_c + ε_c

where gapEffort_c is the country-specific gender gap in attitudes towards academic effort (*THI* or *ATTLNACT*) from model (1b), and Z_c is a development indicator (ESCS, HDI, GDP) or measure of gender norms/stereotypes (Tables 2, 4, S1D, S3B).

Associations between gender gaps in attitudes towards effort and gender gaps in educational outcomes

Model (4b): gapOutcome_c = $\beta_0 + \beta_1$ gapEffort_c + ε_c

where gapEffort_c is the country-specific gender gap in attitudes towards academic effort (*THI* or *ATTLNACT*) from model (1b); and gapOutcome_c is a country-level gender gap in educational outcomes:

- Performance gaps (boys minus girls) in math, reading, or science PISA scores;
- Boys' likelihood of being a low performer relative to girls;
- Share of males among students aged 19-22 enrolled in tertiary education (OECD data);
- Share of males enrolled in tertiary Arts and Humanities or STEM fields;

- Gender gaps in expected years of schooling (UNESCO);
- Gender gaps in intentions to pursue math studies or health-related careers (PISA).

The coefficient β_1 captures the cross-national association between gender gaps in effort attitudes and gender disparities in educational outcomes. Results are reported in Tables 3, S2B-D.

Spatial autocorrelation: We test for spatial dependence in country-level residuals using Moran's I statistic and re-estimate models using spatial error specifications (GS2SLS) when spatial autocorrelation is detected. Spatial diagnostics and spatial error model estimates are reported in Appendix E.

Appendix D: Main Figures and Tables

Figures

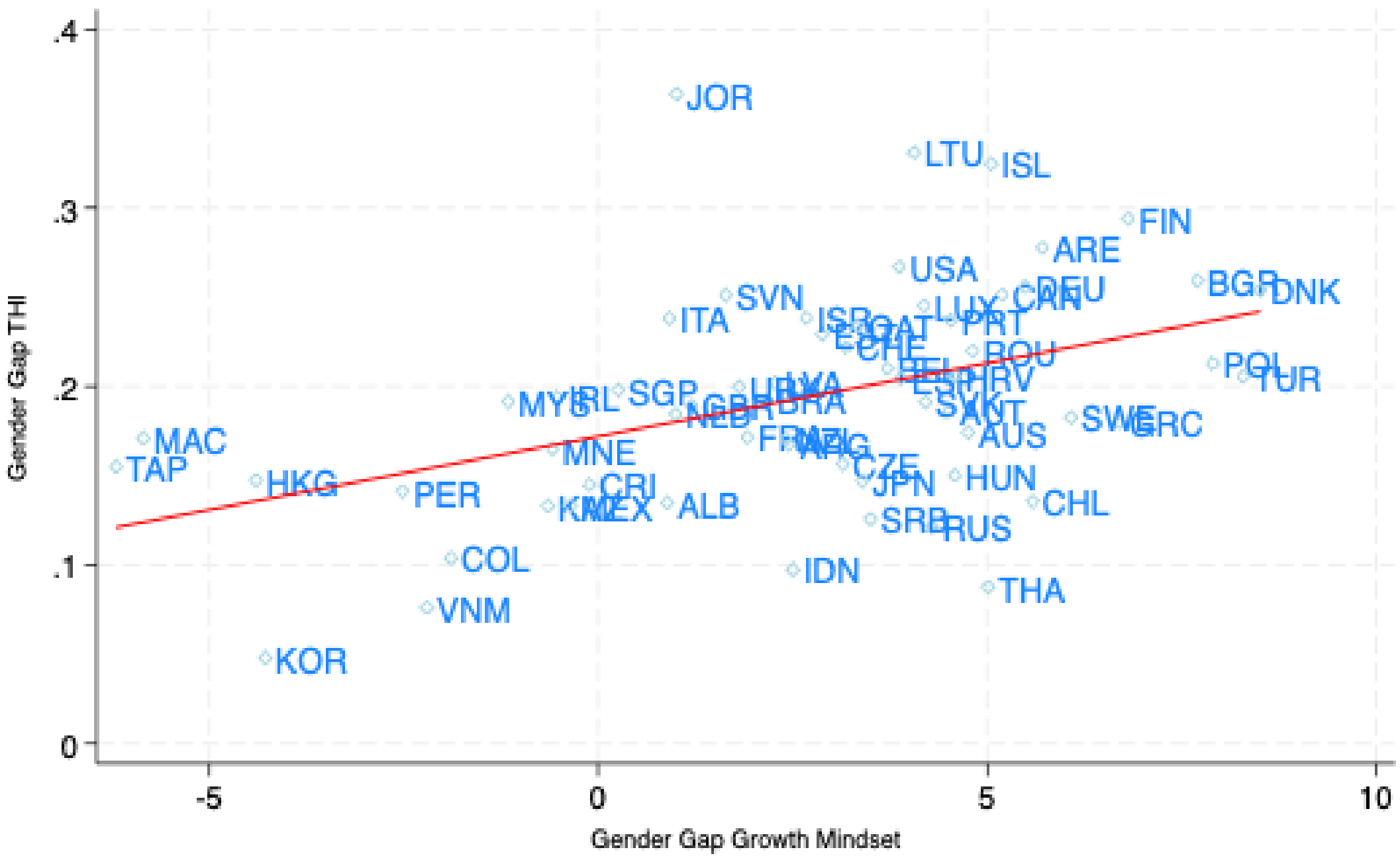

Figure S1. Country-level relationship between gender gaps in attitudes towards academic effort (THI, y axis) and gender differences in growth mindset (x axis). The figure presents the national level of gender gap in attitudes towards trying hard at school (*THI*) relying on PISA data as a function of the national level of gender gap in growth mindset, relying on PISA data. Positive gender gaps in *THI* represent more positive attitudes towards trying hard at school for girls than for boys and positive gender gaps in growth mindset represent a higher growth mindset for girls than for boys. Variables and data sources are described in Appendices A and B. Country codes from ISO3166-1 standard.

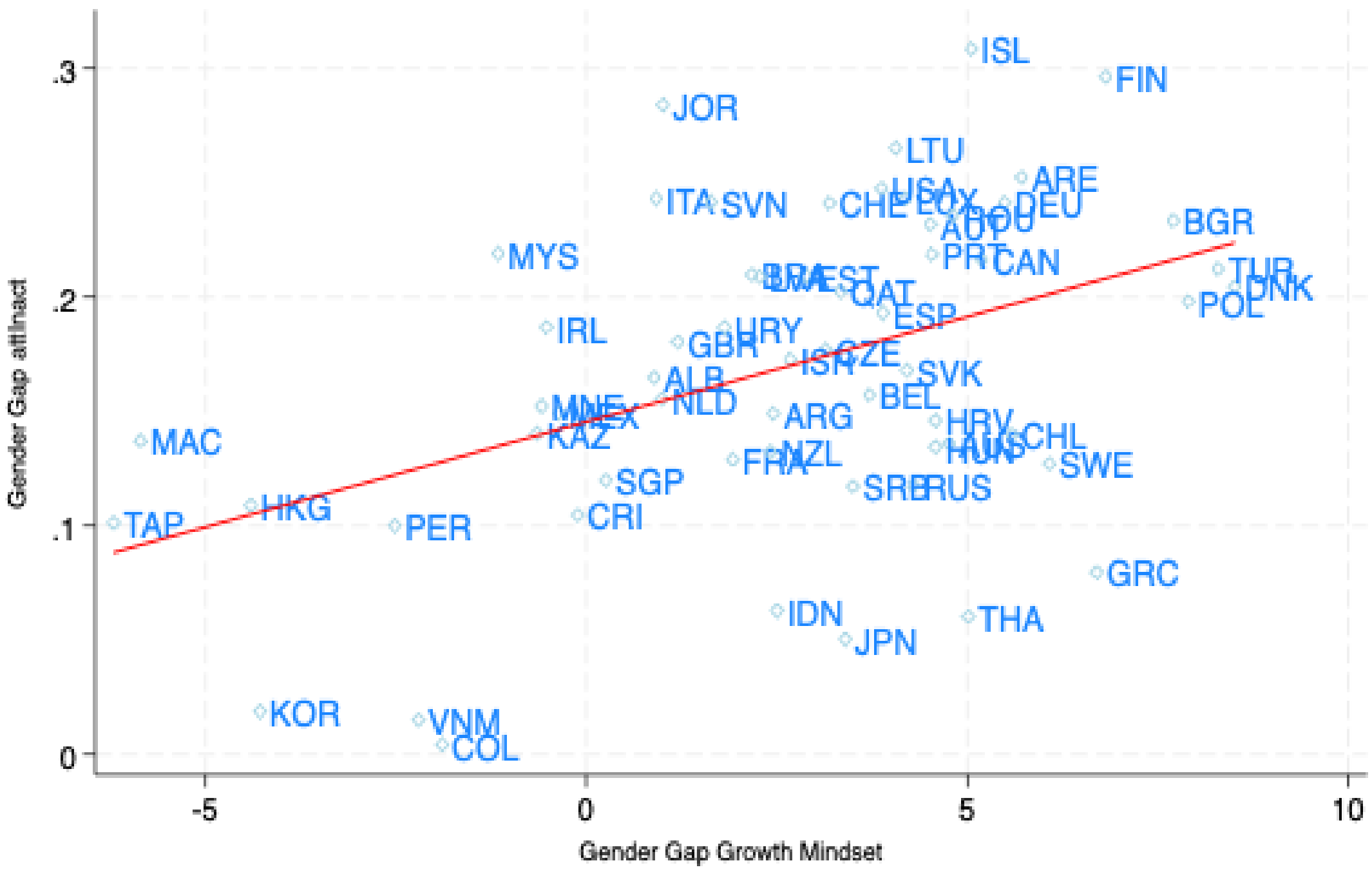

Figure S2: Country-level relationship between gender gaps in attitudes towards academic effort (ATTLNACT, y axis) and gender differences in growth mindset (x axis). The figure presents the national level of gender gap in students' attitudes towards learning activities (*ATTLNACT*), relying on data from PISA, shown as a function of the national level of gender gaps in growth mindset, relying on PISA data. Positive gender gaps in *ATTLNACT* represent more positive attitudes towards learning activities for girls than for boys and positive gender gaps in growth mindset represent a higher growth mindset for girls than for boys. Variables and data sources are described in Appendices A and B. Country codes from ISO3166-1 standard.

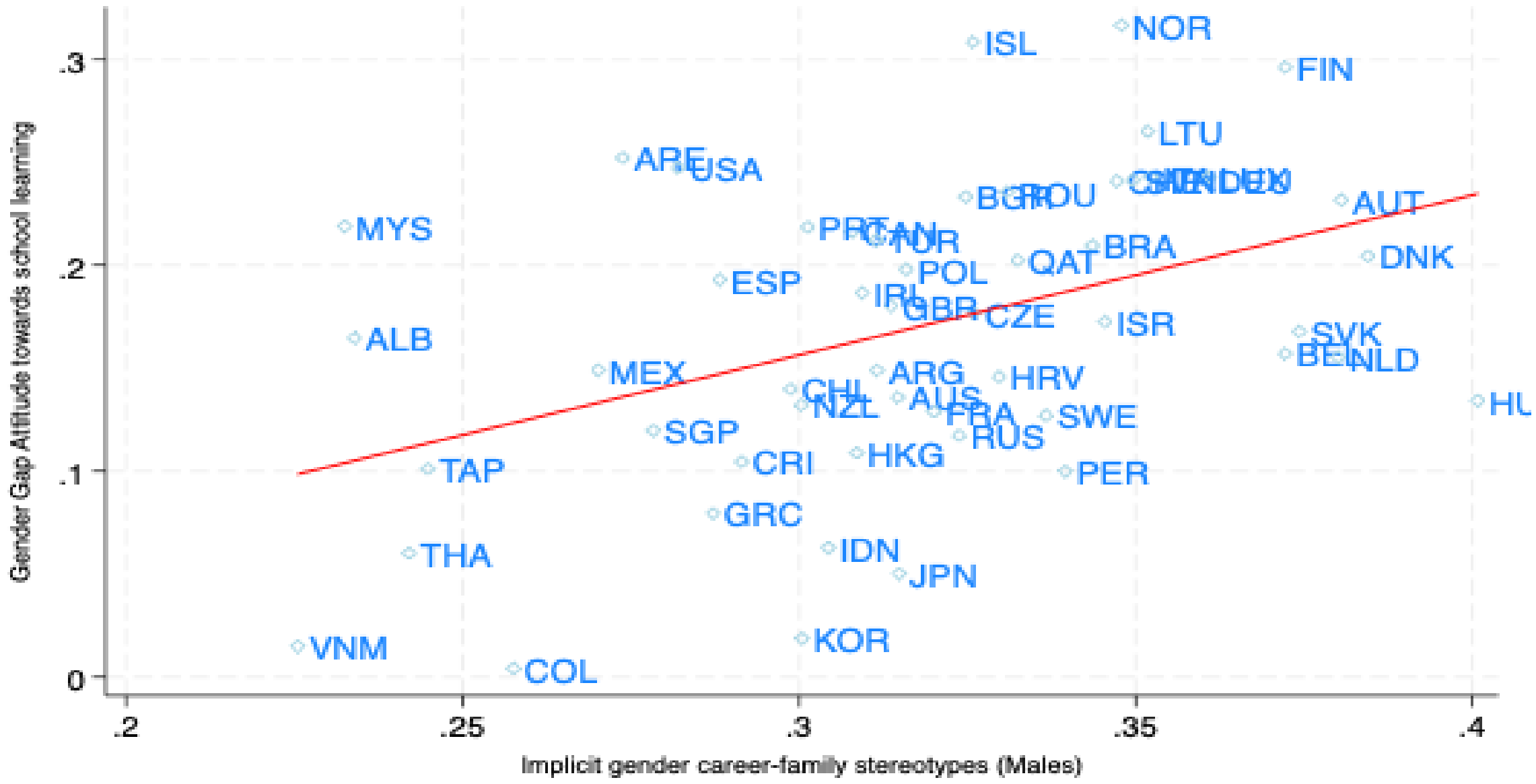

Figure S3: Country-level relationship between gender gaps in attitudes towards academic effort (y axis) and implicit gender career family stereotypes (x axis). The figure presents the national level of gender gap in students' attitudes towards learning activities (*ATTLNACT*), relying on data from PISA, shown as a function of the national level of implicit gender career-family stereotypes among males, relying on Project Implicit (3). Positive gender gaps in *ATTLNACT* represent more positive attitudes towards academic effort for girls than for boys and positive gender career-family stereotypes represent a higher association of men with career and of women with family. Variables and data sources are described in detail in Appendices A and B. Country codes from ISO3166-1 standard.

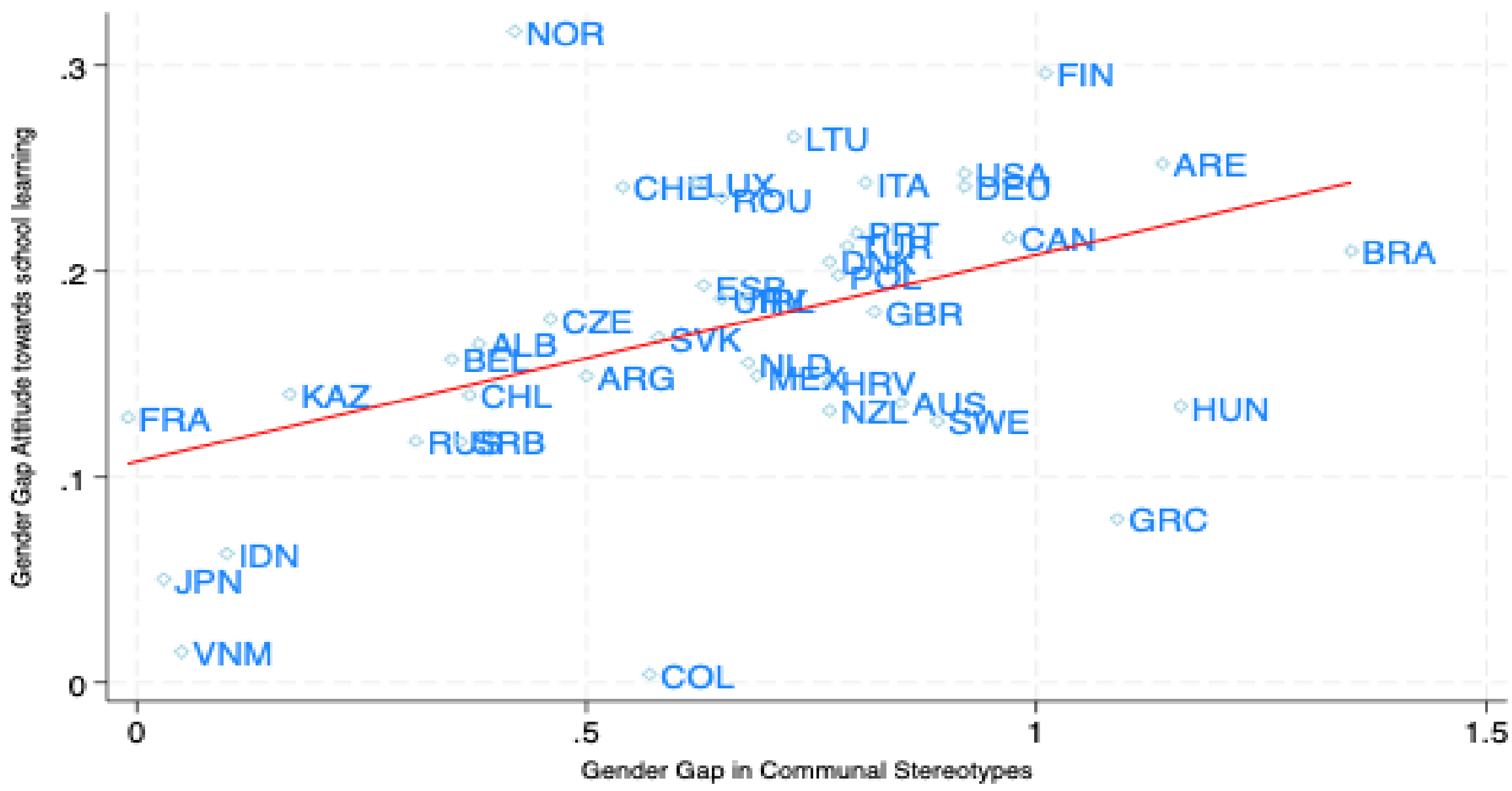

Figure S4: Country-level relationship between gender gaps in attitudes towards academic effort (y axis) and gender stereotypes about communality (x axis). The figure presents the national level of gender gap in students' attitudes towards learning activities (*ATTLNACT*), relying on data from PISA, shown as a function of the national level of gender stereotypes about communality, relying on (6, 7). Positive gender gaps in *ATTLNACT* represent more positive attitudes towards academic effort for girls than for boys and positive gender stereotypes about communality represent a higher rated desirability of communality traits for women than for men. Variables and data sources are described in detail in Appendices A and B. Country codes from ISO3166-1 standard

Tables

Table S1. Gender gaps in attitudes towards academic effort

Table S1A. Gender gaps in attitudes towards academic effort on average and by country in PISA2012, in PISA2018 and in the full sample

A- On the full sample

Panel (i): Attitudes towards trying hard at school (*THI*)

	THI 2012	*THI* 2018	*THI* all
**GIRL**	**0.189*** **	**0.203*** **	**0.196*** **
	(0.00443)	(0.00370)	(0.00265)
Constant	-0.0874***	-0.101***	-0.0938***
	(0.0257)	(0.0166)	(0.0150)
Obs.	300,212	464,249	**764,461**
R-squared	0.00890***	0.0103***	0.00958***

Panel (ii): Attitudes towards learning activities (*ATTLNACT*)

	ATTLNACT 2012	*ATTLNACT* 2018	*ATTLNACT* all
**GIRL**	**0.157*** **	**0.185*** **	**0.171*** **
	(0.00484)	(0.00361)	(0.00272)
Constant	-0.0730***	-0.0923***	-0.0822***
	(0.0224)	(0.0174)	(0.0140)
Obs.	300,720	462,215	**762,935**
R-squared	0.00618***	0.00859***	0.00733***

Notes. The table presents estimates of linear regressions of attitudes towards academic effort on a dummy representing gender (GIRL). Attitudes towards academic effort are captured in Panel (i) by students' agreement with PISA statement '*Trying hard at school is important*' (*THI*) and in Panel (ii) by the PISA index of attitudes towards learning activities (*ATTLNACT*). Both variables are described in Appendix A. Higher *THI* or *ATTLNACT* values correspond to more positive attitudes towards academic effort. In both panels, the first column considers PISA2012, the second column considers PISA2018 and the third column considers the full sample. Variables are standardized within country-year so that coefficients are expressed in within-country-year SD units. Estimation uses senate weights (student weights normalized to sum to one within country-year on the regression sample) and BRR/Fay replicate weights. The model is described in detail in Appendix C: Model (1a). Standard errors in parentheses *** $p<0.01$, ** $p<0.05$, * $p<0.1$

B- By country

Panel (i): Attitudes towards trying hard at school (*THI*)

GENDER GAP *THI*

	2012	2018	ALL	N
AVERAGE	0.189	0,203	0.196	12532
Australia	0.166***	0.182***	0.174***	21505
Austria	0.177***	0.196***	0.187***	9613
Belgium	0.182***	0.238***	0.210***	13143
Canada	0.291***	0.212***	0.251***	34231
Chile	0.102***	0.169***	0.136***	11427
Czech Republic	0.122***	0.191***	0.157***	10083
Denmark	0.293***	0.215***	0.254***	11439
Estonia	0.245***	0.213***	0.229***	8308
Finland	0.302***	0.286***	0.294***	11057
France	0.168***	0.175***	0.172***	8902
Germany	0.247***	0.264***	0.256***	7144
Greece	0.213***	0.146***	0.180***	9524
Hungary	0.140***	0.160***	0.150***	8148
Iceland	0.389***	0.260***	0.325***	5311
Ireland	0.164***	0.223***	0.194***	8798
Israel	0.281***	0.195***	0.238***	9138
Italy	0.242***	0.234***	0.238***	31402
Japan	0.152***	0.142***	0.147***	10198
Korea	0.092**	0.004	0.048**	9953
Luxembourg	0.264***	0.227***	0.245***	8396
Mexico	0.109***	0.158***	0.133***	28095
Netherlands	0.162***	0.207***	0.184***	6651
New Zealand	0.200***	0.141***	0.170***	8720
Norway	0.356***	0.372***	0.364***	8357
Poland	0.236***	0.189***	0.213***	8542
Portugal	0.209***	0.265***	0.237***	9303
Slovak Republic	0.184***	0.199***	0.191***	8611
Slovenia	0.300***	0.203***	0.251***	9790
Spain	0.221***	0.189***	0.205***	50687
Sweden	0.223***	0.142***	0.183***	8199
Switzerland	0.238***	0.206***	0.222***	12757
Turkey	0.187***	0.224***	0.205***	9972
United Kingdom	0.187***	0.191***	0.189***	21289
United States	0.322***	0.212***	0.267***	7870
Albania	0.032	0.237***	0.135***	8810

Argentina	0.175***	0.160***	0.167***	14483
Brazil	0.194***	0.190***	0.192***	20902
Bulgaria	0.206***	0.312***	0.259***	7911
Colombia	0.117***	0.091***	0.104***	12085
Costa Rica	0.136***	0.154***	0.145***	9349
Croatia	0.186***	0.225***	0.205***	9716
Hong Kong-China	0.090**	0.205***	0.148***	8751
Indonesia	0.069*	0.125***	0.097***	15453
Jordan	0.388***	0.340***	0.364***	13111
Kazakhstan	0.074**	0.192***	0.133***	22603
Latvia	0.182***	0.222***	0.202***	7891
Lithuania	0.338***	0.324***	0.331***	9657
Macao-China	0.120***	0.222***	0.171***	7305
Malaysia	0.204***	0.179***	0.191***	9342
Montenegro	0.130***	0.201***	0.165***	9345
Peru	0.146***	0.137***	0.141***	8536
Qatar	0.170***	0.299***	0.234***	18932
Romania	0.118***	0.322***	0.220***	8284
Russian Federation	0.073**	0.169***	0.121***	10613
Serbia	0.080*	0.171***	0.126***	9012
Singapore	0.142***	0.254***	0.198***	10273
Chinese Taipei	0.144***	0.166***	0.155***	11155
Thailand	0.042	0.133***	0.088***	12886
United Arab Emirates	0.303***	0.253***	0.278***	25135
Uruguay	0.188***	0.211***	0.200***	7717
Viet Nam	0.096***	0.056*	0.076***	8641

Panel (ii): Attitudes towards learning activities (*ATTLNACT*)

GENDER GAP *ATTLNACT*

	2012	2018	ALL	N
AVERAGE	0,157	0.185	0.171	12507
Australia	0.124***	0.148***	0.136***	21482
Austria	0.253***	0.211***	0.232***	9595
Belgium	0.087***	0.227***	0.157***	13157
Canada	0.218***	0.214***	0.216***	34201
Chile	0.100***	0.180***	0.140***	11416
Czech Republic	0.123***	0.231***	0.177***	10071

Denmark	0.229***	0.180***	0.205***	11465
Estonia	0.230***	0.188***	0.209***	8297
Finland	0.339***	0.253***	0.296***	11068
France	0.125***	0.132***	0.129***	8888
Germany	0.204***	0.279***	0.241***	7131
Greece	0.094**	0.065**	0.080***	9492
Hungary	0.116***	0.152***	0.134***	8135
Iceland	0.381***	0.235***	0.308***	5304
Ireland	0.149***	0.225***	0.187***	8790
Israel	0.189***	0.156***	0.173***	9090
Italy	0.222***	0.264***	0.243***	31382
Japan	0.025	0.076***	0.050**	10191
Korea	0.057	-0.019	0.019	9946
Luxembourg	0.250***	0.236***	0.243***	8388
Mexico	0.104***	0.195***	0.149***	28077
Netherlands	0.095*	0.216***	0.155***	6657
New Zealand	0.157***	0.107***	0.132***	8711
Norway	0.326***	0.307***	0.317***	8393
Poland	0.232***	0.165***	0.198***	8536
Portugal	0.159***	0.278***	0.219***	9293
Slovak Republic	0.167***	0.169***	0.168***	8592
Slovenia	0.289***	0.194***	0.241***	9783
Spain	0.207***	0.179***	0.193***	50549
Sweden	0.189***	0.066**	0.127***	8193
Switzerland	0.256***	0.226***	0.241***	12755
Turkey	0.192***	0.233***	0.212***	9951
United Kingdom	0.165***	0.196***	0.180***	21275
United States	0.283***	0.212***	0.247***	7863
Albania	0.031	0.298***	0.165***	8774
Argentina	0.134***	0.164***	0.149***	14372
Brazil	0.197***	0.223***	0.210***	20861
Bulgaria	0.190***	0.277***	0.233***	7866
Colombia	-0.003	0.011	0.004	12052
Costa Rica	0.074*	0.135***	0.105***	9329
Croatia	0.096**	0.196***	0.146***	9691
Hong Kong-China	0.061	0.157***	0.109***	8744
Indonesia	0.028	0.098***	0.063***	15398
Jordan	0.301***	0.267***	0.284***	13043
Kazakhstan	0.083**	0.198***	0.140***	22499
Latvia	0.182***	0.235***	0.209***	7883
Lithuania	0.333***	0.197***	0.265***	9637
Macao-China	0.103***	0.171***	0.137***	7307
Malaysia	0.260***	0.178***	0.219***	9336

Montenegro	0.114***	0.190***	0.152***	9310
Peru	0.066*	0.134***	0.100***	8526
Qatar	0.118***	0.287***	0.202***	18827
Romania	0.124***	0.347***	0.236***	8242
Russian Federation	0.082**	0.153***	0.117***	10574
Serbia	0.050	0.184***	0.117***	8962
Singapore	0.081**	0.159***	0.120***	10263
Chinese Taipei	0.088**	0.114***	0.101***	11145
Thailand	0.043	0.077***	0.060***	12865
United Arab Emirates	0.265***	0.240***	0.252***	25012
Uruguay	0.159***	0.214***	0.187***	7671
Viet Nam	0.000	0.030	0.015	8629

Notes. The table presents estimates of the gender gaps by country in attitudes towards academic effort, as well as the number of observations by country. Attitudes towards academic effort are captured in the first panel by students' agreement with PISA statement *'Trying hard at school is important'* (*THI*) and in the second panel by the PISA index of attitudes towards learning activities (*ATTLNACT*). Both variables are described in Appendix A. The first two columns present the gender gaps for PISA2012 and PISA2018, while the last two columns present the gender gap and the number of observations, relying on both surveys. Positive gender gaps correspond to more positive attitudes towards academic effort among girls than among boys. The first line provides the average gender gaps and number of observations across countries. Variables are standardized within country-year so that coefficients are expressed in within-country-year SD units. Estimation uses senate weights (student weights normalized to sum to one within country-year on the regression sample) and BRR/Fay replicate weights. The model is described in Appendix C: Models (1b) and (1c). *** $p<0.01$, ** $p<0.05$, * $p<0.1$

Table S1B. Gender gaps in attitudes towards academic effort with controls

Panel (i): Attitudes towards trying hard at school (*THI*)

	Dependent Variable is THI				
**GIRL**	**0.191*** **	**0.185*** **	**0.174*** **	**0.185*** **	**0.174*** **
	(0.00273)	(0.00281)	(0.00296)	(0.00282)	(0.00299)
Constant	-0.0909***	-0.0889***	-0.0831***	-0.0887***	-0.0801***
	(0.0173)	(0.0171)	(0.0161)	(0.0171)	(0.0161)
Obs.	685,115	685,115	623,066	685,115	623,066
R-squared	0.00927***	0.0181***	0.0675***	0.0182***	0.0680***
CONTROLS	NO CONTROL	BASIC CONTROLS	BASIC and Additional CONTROLS	BASIC CONTROLS & interaction with gender	BASIC and Additional CONTROLS &

					interaction with gender

Panel (ii): Attitudes towards learning activities (*ATTLNACT*)

	Dependent Variable is ATTLNACT				
**GIRL**	**0.167*****<br>(0.00281)	**0.161*****<br>(0.00280)	**0.150*****<br>(0.00284)	**0.161*****<br>(0.00282)	**0.150*****<br>(0.00289)
Constant	-0.0760***<br>(0.0182)	-0.0744***<br>(0.0180)	-0.0700***<br>(0.0175)	-0.0741***<br>(0.0179)	-0.0684***<br>(0.0174)
Obs.	683,876	683,876	622,414	683,876	622,414
R-squared	0.00711***	0.0204***	0.0916***	0.0205***	0.0918***
CONTROLS	NO CONTROL	BASIC CONTROLS	BASIC and Additional CONTROLS	BASIC CONTROLS & interaction with gender	BASIC and Additional CONTROLS & interaction with gender

Notes. The table presents estimates of linear regressions of attitudes towards academic effort on a dummy representing gender (GIRL) with various controls. Attitudes towards academic effort are captured in Panel (i) by students' agreement with PISA statement *'Trying hard at school is important'* (*THI*) and in Panel (ii) by the PISA index of attitudes towards learning activities (*ATTLNACT*). Higher *THI* or *ATTLNACT* values correspond to more positive attitudes towards academic effort. In column (2), basic controls include the socioeconomic level of the student (ESCS), an indicator of their home education resources (HEDRES), their age, indicators of parental education (MISCED, FISCED, HISCED, PARED), the grade they are in (Grade), whether they have repeated a grade during their studies (Repeat) and in column (3) additional controls include the student's sense of belonging (BELONG), and indicators of their truancy (Late, skip day, skip class). Columns (4) and (5) consider the same controls as in columns (2) and (3) adding their interaction with gender. All variables are described in Appendix A. Variables are standardized within country-year. Estimation uses senate weights (student weights normalized to sum to one within country-year on the regression sample) and BRR/Fay replicate weights. The model is described in Appendix C Model (1d). Standard errors in parentheses *** $p<0.01$, ** $p<0.05$, * $p<0.1$

Table S1C. Gender gaps in attitudes towards academic effort for various subgroups

A- Various subgroups

Panel (i): Attitudes towards trying hard at school (*THI*)

THI	**All**	**OECD**	**No OECD**	**Modal grade**	**High grade**	**Low grade**	**No Repeat**	**Repeat**
**GIRL**	**0.196***	**0.209***	**0.180***	**0.196***	**0.144***	**0.182***	**0.199***	**0.192***

	(0.0026)	(0.00393)	(0.00433)	(0.00330)	(0.0292)	(0.0166)	(0.0163)	(0.00296)
Cstt	-0.0938***	-0.101***	-0.0896*	-0.0852**	-0.207	-0.0945***	-0.0864***	-0.344***
	(0.0150)	(0.0113)	(0.0496)	(0.0354)	(0.191)	(0.0350)	(0.0320)	(0.103)
Obs.	764,461	446,563	317,898	560,790	59,239	144,432	674,557	89,904
R-sq.	0.0096***	0.0107***	0.00851	0.0106*	0.0132**	0.0126*	0.00986*	0.0131*

THI	**Low ESCS**	**High ESCS**	**Low Truancy**	**High Truancy**	**High FISCED**	**Low FISCED**	**High MISCED**	**Low MISCED**
**GIRL**	**0.205*****	**0.189*****	**0.171*****	**0.209*****	**0.190*****	**0.219*****	**0.204*****	**0.167*****
	(0.00363)	(0.00392)	(0.00322)	(0.00455)	(0.00592)	(0.0260)	(0.00613)	(0.0291)
Cstt	-0.0893***	-0.0962***	0.00150	-0.305***	-0.0999	-0.113	-0.176***	0.0591
	(0.0240)	(0.0360)	(0.0311)	(0.0334)	(0.0633)	(0.0749)	(0.0439)	(0.0735)
Obs.	363,834	400,627	463,145	301,316	217,132	64,081	223,508	61,741
R-sq.	0.0114**	0.0105***	0.00850*	0.0120***	0.0128**	0.0121	0.0135***	0.00932

THI	**Absent father**	**Absent mother**	**Present father**	**Both parents at home**
**GIRL**	**0.205*****	**0.205*****	**0.182*****	**0.181*****
	(0.0129)	(0.0246)	(0.00546)	(0.00560)
Cstt	-0.268***	-0.353***	-0.0429	-0.0372
	(0.0750)	(0.0931)	(0.0319)	(0.0334)
Obs.	42,466	16,652	233,341	225,859
R-sq.	0.0114***	0.0168**	0.00890***	0.00894***

THI	**Anglo.**	**West. Europe**	**Nordics**	**South Europe**	**CEE**	**East Asia**	**Latin Am.**	**Middle East**
**GIRL**	**0.208*****	**0.211*****	**0.284*****	**0.213*****	**0.193*****	**0.132*****	**0.152*****	**0.278*****
	(0.00763)	(0.00827)	(0.00973)	(0.00944)	(0.00549)	(0.00772)	(0.00821)	(0.00936)
Cstt	-0.101***	-0.107***	-0.143***	-0.106***	-0.0925***	-0.0625***	-0.0786***	-0.142***
	(0.0111)	(0.0163)	(0.0147)	(0.0118)	(0.0155)	(0.0161)	(0.0149)	(0.0124)
Obs.	102,413	66,606	44,363	110,888	157,324	103,957	112,594	66,316
R-sq.	0.0108***	0.0111***	0.0201***	0.0113***	0.00931***	0.00434***	0.00578***	0.0193***

Panel (ii): Attitudes towards learning activities (*ATTLNACT*)

ATT.	**All**	**OECD**	**No OECD**	**Modal grade**	**High grade**	**Low grade**	**Repeat**	**No Repeat**
**GIRL**	**0.171*****	**0.187*****	**0.152*****	**0.171*****	**0.120*****	**0.150*****	**0.165*****	**0.167*****
	(0.0027)	(0.00379)	(0.00444)	(0.00330)	(0.0262)	(0.0174)	(0.0164)	(0.00273)
Cstt	-0.0822***	-0.0913***	-0.0730***	-0.0746***	-0.189	-0.0838***	-0.452***	-0.0671***
	(0.0140)	(0.00909)	(0.0146)	(0.0173)	(0.164)	(0.0263)	(0.104)	(0.0145)
Obs.	762,935	446,117	316,818	559,911	59,164	143,860	89,457	673,478
R-sq.	0.0073***	0.00867***	0.00619	0.00837*	0.0128***	0.0123*	0.00764*	0.0132

ATT.	Low ESCS	High ESCS	Low Truancy	High Truancy	High FISCED	Low FISCED	High MISCED	Low MISCED
**GIRL**	**0.181*** **	**0.165*** **	**0.150*** **	**0.178*** **	**0.163*** **	**0.175*** **	**0.177*** **	**0.157*** **
	(0.00406)	(0.00367)	(0.00312)	(0.00503)	(0.00604)	(0.0229)	(0.00607)	(0.0280)
Cstt	-0.0938***	-0.0768***	0.0256*	-0.324***	-0.0519	-0.125*	-0.0956***	-0.0772
	(0.0223)	(0.0169)	(0.0154)	(0.0286)	(0.0330)	(0.0678)	(0.0358)	(0.0754)
Obs.	362,921	400,014	462,694	300,241	216,679	63,931	223,004	61,587
R-sq.	0.00964**	0.00856***	0.00643*	0.0105***	0.0105***	0.0110	0.0113***	0.00909

ATT.	Absent father	Absent mother	Present father	Both parents at home
**GIRL**	**0.174*** **	**0.148*** **	**0.152*** **	**0.152*** **
	(0.0125)	(0.0240)	(0.00571)	(0.00582)
Cstt	-0.260***	-0.347***	-0.0292	-0.0223
	(0.0708)	(0.0946)	(0.0238)	(0.0240)
Obs.	42,541	16,675	233,743	226,243
R-sq.	0.00951**	0.0145	0.00624***	0.00632***

ATT	(1) Anglo.	(2) West. Europe	(3) Nordics	(4) South Europe	(5) CEE	(6) East Asia	(7) Latin Am.	(8) Middle East
**GIRL**	**0.183*** **	**0.200*** **	**0.251*** **	**0.189*** **	**0.182*** **	**0.089*** **	**0.130*** **	**0.228*** **
	(0.00806)	(0.00858)	(0.0100)	(0.00903)	(0.00596)	(0.00782)	(0.00772)	(0.0107)
Cstt	-0.0893***	-0.101***	-0.126***	-0.0939***	-0.0872***	-0.0424***	-0.0673***	-0.116***
	(0.0110)	(0.0184)	(0.0142)	(0.0109)	(0.0151)	(0.0153)	(0.0167)	(0.0144)
Obs.	102,322	66,571	44,423	110,667	156,852	103,824	112,304	65,972
R-sq.	0.00838***	0.00997***	0.0157***	0.00896***	0.00825***	0.00199***	0.00424***	0.0129***

Notes. The table presents estimates of linear regressions of attitudes towards academic effort on a dummy representing gender (GIRL) for selected subgroups. Attitudes towards academic effort is captured in Panel (i) by students' agreement with PISA statement *'Trying hard at school is important'* (*THI)* and in Panel (ii) by the PISA index of attitudes towards learning activities (*ATTLNACT*). Higher *THI* or *ATTLNACT* values correspond to more positive attitudes towards academic effort. Both variables are standardized within country-year. The table considers the restriction (i) to OECD and non-OECD countries, (ii) to students who are in modal grade, in lower and in higher grades, (iii) to students who have never repeated a grade and to students who have repeated at least one grade (iv) to students from low and high socioeconomic background, (v) to students with low or high level of truancy, (vi) to students with father (FISCED) or mother (MISCED) with high or low level of education, (vii) to students with father or mother not at home, with father present at home and with both parents at home, (viii) to countries belonging to the eight following cultural regions 1. Anglophone (United States, Canada, United Kingdom, Ireland, Australia, New Zealand) 2 West Continental Europe (Belgium, Netherlands, Luxembourg, Germany, Austria, Switzerland, France) 3. Nordics (Denmark, Sweden, Norway, Finland, Iceland) 4. Southern/Mediterranean Europe (Spain, Portugal, Italy, Greece, Turkey) 5. CEE/Balkans/Baltics (Poland, Czech Republic, Slovak Republic, Slovenia, Croatia, Hungary, Romania, Bulgaria, Serbia, Albania, Kazakhstan, Montenegro, Estonia, Latvia, Lithuania, Russian Federation) 6. East/Southeast Asia (Japan, Korea, Hong Kong, Macao, Singapore, Chinese Taipei, Thailand, Malaysia, Indonesia, Vietnam) 7. Latin America (Mexico, Chile, Argentina, Brazil, Colombia, Costa Rica, Peru, Uruguay 8. Middle East/Central Asia (Israël, Jordan, Qatar, United Arab Emirates). Description of all variables and definitions for low and high levels of socioeconomic status, truancy, education are provided in Appendix A. Estimation uses senate weights (student weights normalized to

sum to one within country-year on the regression sample) and BRR/Fay replicate weights. The model is described in the Methods section in Appendix C (Model (1v)). Standard errors in parentheses *** *p<0.01*, ** *p<0.05*, * *p<0.1*

Table S1D. The Gender Equality Paradox for attitudes towards academic effort.

A. Contemporary values

Panel (i) Attitudes towards trying hard at school (*THI*)

	Dependent variable is Gender gap in THI				
**ESCS**	**0.545*** **				
	(0.0936)				
**HDI**		**0.397** **			
		(0.153)			
**lnGDP**			**0.417*** **		
			(0.131)		
**GGI**				**0.345***	
				(0.193)	
**Indiv.**					**0.429*** **
					(0.125)
Cst.	7.88e-11	8.05e-10	2.14e-09	-0	2.71e-09
	(0.108)	(0.121)	(0.118)	(0.127)	(0.125)
obs.	61	59	60	56	53
R-squared	0.297	0.158	0.174	0.119	0.184

Panel (ii) Attitudes towards learning activities (*ATTLNACT*)

	Dependent variable is Gender gap in ATTLNACT				
**ESCS**	**0.478*** **				
	(0.117)				
**HDI**		**0.351** **			
		(0.149)			
**lnGDP**			**0.362*** **		
			(0.132)		
**GGI**				**0.355** **	
				(0.166)	
**Indiv.**					**0.413*** **
					(0.133)
Cst.	2.32e-09	1.44e-10	-4.89e-09	1.65e-09	6.87e-11
	(0.113)	(0.123)	(0.121)	(0.126)	(0.126)
obs.	61	59	60	56	53
R-squared	0.229	0.123	0.131	0.126	0.170

B. Historical Values

Panel (i) Attitudes towards trying hard at school (*THI*)

	Dependent Variable is Gender Gap in THI				
**GDP 1960**	**0.689*****				
	(0.141)				
**GDP 1970**		**0.684*****			
		(0.134)			
**GDP 1980**			**0.510*****		
			(0.139)		
**HDI 1990**				**0.413*****	
				(0.135)	
**Parasite Stress (M)**					**-0.507*****
					(0.0981)
Constant	-7.20e-10	2.44e-09	-3.12e-09	-2.33e-09	3.38e-09
	(0.132)	(0.127)	(0.138)	(0.128)	(0.115)
Observations	31	34	40	52	57
R-squared	0.474	0.468	0.261	0.171	0.257

Panel (ii) Attitudes towards learning activities (*ATTLNACT*)

	Dependent Variable is Gender Gap in ATTLNACT				
**GDP 1960**	**0.559*****				
	(0.148)				
**GDP 1970**		**0.536*****			
		(0.148)			
**GDP 1980**			**0.435*****		
			(0.140)		
**HDI 1990**				**0.356****	
				(0.146)	
**Parasite Stress (M)**					

					**-0.499*****<br>(0.131)
Constant	2.97e-10<br>(0.151)	5.00e-09<br>(0.147)	6.96e-09<br>(0.144)	-6.63e-09<br>(0.131)	4.54e-09<br>(0.116)
Observations	31	34	40	52	57
R-squared	0.313	0.288	0.190	0.127	0.249

Notes. The table presents estimates at the country level of linear regressions of gender gaps (Girls-Boys) in attitudes towards academic effort on indicators of country level of development (Human Development Index, ESCS, Gross Domestic Product), of gender equality (Gender Gap Index), of Individualism (Hofstede's individualism). Section A considers values of such indicators that are contemporary to the PISA surveys while Section B considers historical values, such as Gross Domestic Product (GDP) in 1960, in 1970, in 1980 or Human Development Index (HDI) in 1990. It also considers (the opposite of) an indicator of country level of Parasite Stress, using data from (2). Attitudes towards academic effort are captured in Panel (i) by students' agreement with PISA statement *'Trying hard at school is important'* (*THI*) and in Panel (ii) by the PISA index of attitudes towards learning activities (*ATTLNACT*). Positive gender gaps correspond to more positive attitudes towards academic effort among girls than among boys. All the variables and data sources are described in Appendices A-B. All variables are standardized within the regression sample so that the coefficients in the regression represent the change in SD units of the dependent variable associated with a 1-SD increase in the explanatory variable and are equivalent to Pearson correlation coefficients. The model is described in Appendix C (Model (3a)). Robust standard errors in parentheses *** *p<0.01,* ** *p<0.05,* * *p<0.1*

Table S1E - Robustness checks: link between attitudes towards academic effort and ESCS

Panel A : THI

Specification	Outcome	Female	Female × ESCS	N	Notes
(i) Design-consistent (PISA repest)	THI (std)	0.175***<br>(0.003)	**0.045*****<br>(0.004)	764,461	Main specification
(ii) Clustered SEs	THI (std)	0.175***<br>(0.007)	**0.045*****<br>(0.008)	764,461	Conservative SEs
(iii) Multilevel model	THI (std)	0.175***<br>(0.007)	**0.045*****<br>(0.008)	764,461	Random intercepts
(iv) Non-standardized + gender specific slopes	THI (raw)	0.172***<br>(0.003)	**0.043*****<br>(0.004)<br>boys: b=-0.04<br>girls: b=0	764,461	No standardization<br>No country FE
(v) Unweighted + Clustered SEs	THI (std)	0.178***<br>(0.007)	**0.041*****<br>(0.007)	764,461	No survey weights
(viA) With individual controls (A)	THI (std)	0.166***<br>(0.003)	**0.042*****<br>(0.004)	716,419	SES, parents, etc.

Specification	Outcome	Female	Female × ESCS	N	Notes
(viB) With individual controls (B)	THI (std)	0.165*** (0.003)	**0.052***** (0.004)	694,353	+ belonging

Panel B: ATTLNACT

Specification	Outcome	Female	Female × ESCS	N	Notes
(i) Design-consistent (PISA repest)	ATTLNACT (std)	0.151*** (0.003)	**0.043***** (0.004)	762,935	Main specification
(ii) Clustered SEs	ATTLNACT (std)	0.151*** (0.009)	**0.043***** (0.010)	762,935	Conservative SEs
(iii) Multilevel model	ATTLNACT (std)	0.151*** (0.009)	**0.043***** (0.010)	762,935	Random intercepts
(iv) Non-standardized + gender specific slopes	ATTLNACT (raw)	0.149*** (0.003)	**0.044***** (0.004) boys: b=-0.015 girls: b=0.029	762,935	No standardization No country FE
(v) Unweighted + Clustered SEs	ATTLNACT (std)	0.158*** (0.009)	**0.036***** (0.009)	762,935	No survey weights
(viA) With individual controls (A)	ATTLNACT (std)	0.142*** (0.003)	**0.042***** (0.004)	715,111	SES, parents, etc.
(viB) With individual controls (B)	ATTLNACT (std)	0.140*** (0.003)	**0.055***** (0.004)	693,456	+ belonging

Notes: All specifications (i)-(vi) correspond to model (2) and are described in detail in Appendix C. Country and year fixed effects are included. THI (std) and ATTLNACT (std) = attitudes toward trying hard, standardized within country-year. ESCS = PISA Economic, Social and Cultural Status index. Standard errors in parentheses. Models (i), (iv), (vi) use PISA replicate weights (BRR); Model (ii) uses analytic weights with cluster-robust standard errors; Model (iii) uses maximum likelihood with robust SEs; Model (v) uses no weights and cluster-robust standard errors. Model (viA) includes grade repetition, parental education, student-level ESCS, home educational resources as individual controls (repeat pared hisced escs hedres) and model (viB) adds sense of belonging at school (belong). All items are described in Appendix A. *** *p<0.01,* ** *p<0.05,* * *p<0.1*

Table S2A. Link at the individual level between Academic Performance and Attitudes towards academic effort

Panel (i) Attitudes towards trying hard at school (*THI*)

THI	General Perf.	Math Perf.	Reading Perf.	Science Perf.	Likelihood of low performer	Likelihood of

					in general	low performer in reading
**GIRLS**						
THI	0.0541***	0.0439***	0.0616***	0.0479***	-0.0225***	-0.0275***
	(0.00233)	(0.00220)	(0.00244)	(0.00243)	(0.00102)	(0.00103)
**BOYS**						
THI	0.0588***	0.0460***	0.0690***	0.0513***	-0.0224***	-0.0238***
	(0.00240)	(0.00221)	(0.00254)	(0.00233)	(0.00109)	(0.00109)
**ALL**						
Girl**THI*	-0.00462	-0.00232	-0.00716**	-0.00320	-0.000145	-0.00388**
	(0.00314)	(0.00300)	(0.00321)	(0.00312)	(0.00148)	(0.00152)

Panel (ii) Attitudes towards learning activities (*ATTLNACT*)

ATTLNACT	General Perf.	Math Perf.	Reading Perf.	Science Perf.	Likelihood of low performer in general	Likelihood of low performer in reading
**GIRLS**						
ATTLNACT	0.0805***	0.0694***	0.0863***	0.0728***	-0.0348***	-0.0388***
	(0.00222)	(0.00212)	(0.00230)	(0.00229)	(0.00102)	(0.000974)
**BOYS**						
ATTLNACT	0.0872***	0.0731***	0.0966***	0.0775***	-0.0347***	-0.0365***
	(0.00245)	(0.00231)	(0.00260)	(0.00239)	(0.00115)	(0.00114)
**ALL**						
Girl* *ATTLNACT*	-0.00717**	-0.00441	-0.0107***	-0.00507	0.000141	-0.00219
	(0.00315)	(0.00307)	(0.00324)	(0.00310)	(0.00158)	(0.00148)

Panel (iii) Differences across domains

	**THI**	**ATTLNACT**
Reading − Mathematics	0.043***	0.040***
	(0.0009)	(0.0009)

	THI	ATTLNACT
Reading − Science	0.033***	0.031***
	(0.0008)	(0.0008)
Science − Mathematics	0.010***	0.009***
	(0.0008)	(0.0008)

Notes. The table presents estimates of linear regressions at the individual level of indicators of academic performance on attitudes towards academic effort. Attitudes towards academic effort are captured in Panel (i) by students' agreement with PISA statement *'Trying hard at school is important'* (*THI*) and in Panel (ii) by the PISA index of attitudes towards learning activities (*ATTLNACT*). Higher *THI* or *ATTLNACT* values correspond to more positive attitudes towards academic effort. Academic performance is captured by the aggregate score in the three domains (general performance), by the score in mathematics, reading and science separately, by the likelihood of being a low performer (i.e., performing below average in their country in the given PISA cycle) in aggregate or in reading only. Results are presented for girls only, for boys only, and for the gender differences; the "Girl × THI" (or "Girl × ATTLNACT") coefficient represents the interaction term testing whether the association between effort attitudes and performance differs by gender (i.e., the difference between girls' and boys' slopes). Panel (iii) reports differences across domains in the association between effort attitudes and performance, estimated from a separate pooled model in which the three domain-specific scores (reading, mathematics, science) are stacked, with domain × attitude interaction terms, country and year fixed effects, and standard errors clustered at the student level (PV1 scores); a positive coefficient indicates a stronger association in the first domain than in the second. Description of all variables are provided in Appendix A. All variables are standardized within country-year. Estimation uses senate weights (student weights normalized to sum to one within country-year on the regression sample) and BRR/Fay replicate weights. The models are described in Appendix C (Models (3a) and (3a')). Standard errors in parentheses *** $p<0.01$, ** $p<0.05$, * $p<0.1$

Table S2B. Country-level analysis. Link between gender gaps in attitudes towards academic effort and gender gaps in academic performance (G-B)

Panel (i) Attitudes towards trying hard at school (*THI*)

	**Gender gap (G-B) in ...**					
VARIABLES	**Gen. Score**	**Math score**	**Reading score**	**Science score**	**Likelihood low performer**	**Likelihood low perf. in reading**
**Gap *THI***	**0.433*** **	**0.350** **	**0.463*** **	**0.403** **	**-0.407*** **	**-0.473*** **
	(0.151)	(0.143)	(0.144)	(0.161)	(0.151)	(0.139)
Constant	6.67e-10	1.72e-09	-4.37e-10	1.25e-09	-3.97e-09	-6.22e-09
	(0.116)	(0.121)	(0.114)	(0.118)	(0.118)	(0.114)
Obs.	61	61	61	61	61	61
R-squared	0.188	0.123	0.214	0.163	0.166	0.224

Panel (ii) Attitudes towards learning activities (*ATTLNACT*)

VARIABLES	Gender gap (G-B) in ... Gen. Score	Math score	Reading score	Science score	Likelihood low performers	Likelihood low perf. in reading
**Gap *ATTLNACT***	**0.384*** **	**0.305** **	**0.417*** **	**0.360** **	**-0.376*** **	**-0.451*** **
	(0.141)	(0.144)	(0.140)	(0.142)	(0.137)	(0.127)
Constant	-1.81e-10	1.05e-09	-1.36e-09	4.52e-10	-3.14e-09	-5.22e-09
	(0.119)	(0.123)	(0.117)	(0.120)	(0.120)	(0.115)
Obs.	61	61	61	61	61	61
R-squared	0.147	0.093	0.174	0.130	0.141	0.204

Notes. The table presents estimates at the country level (N=61) of linear regressions of gender gaps in academic performance on gender gaps in attitudes towards academic effort. Academic performance is captured by the score in mathematics, reading and science, by the likelihood of being a low performer (i.e., performing below average in their country) in aggregate or in reading only. Attitudes towards academic effort are captured in Panel (i) by students' agreement with PISA statement *'Trying hard at school is important'* (*THI*) and in Panel (ii) by the PISA index of attitudes towards learning activities (*ATTLNACT*). Positive gender gaps correspond to more positive attitudes towards academic effort among girls than among boys. All the variables and data sources are described in Appendix A. Country gender gaps in performance are computed like the country gender gaps in attitudes towards academic effort, using model (1b) in Appendix C. All gender gaps are standardized within the regression sample so that the coefficients in the regression represent the change in SD units of the dependent variable associated with a 1-SD increase in the explanatory variable and are equivalent to Pearson correlation coefficients. The model is described in Appendix C (Model (3b)). Robust standard errors in parentheses *** *p<0.01*, ** *p<0.05*, * *p<0.1*

Table S2C. Country-level analysis. Link between gender gaps in attitudes towards academic effort and gender differences in tertiary education or in expected years of schooling

Panel (i) Attitudes towards trying hard at school (*THI*)

VARIABLES	Male enrollment in tertiary education (1)	Male enrollment in tertiary education (2)	Gender Gap in expected years of schooling
**Gender gap *THI***	-0.430**	**-0.512** **	**0.354*** **
	(0.163)	(0.205)	(0.122)
Constant	0.104	0.119	-0.0148
	(0.150)	(0.165)	(0.122)

Observations	40	39	59
R-squared	0.156	0.227	0.121

Panel (ii) Attitudes towards learning activities (*ATTLNACT*)

VARIABLES	**Male enrollment in tertiary education (1)**	**Male enrollment in tertiary education (2)**	**Gender Gap in expected years of schooling**
**Gender gap *ATTLNACT***	-0.335**	**-0.498****	**0.355*****
	(0.159)	(0.199)	(0.112)
Constant	0.0886	0.135	-0.0191
	(0.171)	(0.166)	(0.123)
Observations	40	39	59
R-squared	0.099	0.225	0.118

Notes. The table presents estimates at the country level of linear regressions of gender gaps in tertiary education or expected years of schooling on gender gaps in attitudes towards academic effort. Attitudes towards academic effort are captured in Panel (i) by students' agreement with PISA statement *'Trying hard at school is important'* (*THI*) and in Panel (ii) by the PISA index of attitudes towards learning activities (*ATTLNACT*). Positive gender gaps correspond to more positive attitudes towards academic effort among girls than among boys. Gender differences in tertiary education are captured by the share of males among students aged 19-22 enrolled in tertiary education in 2017 including Israel in col. (1) and excluding Israel in col. (2). The gender gap (Girls-Boys) in expected years of schooling (EYS) is considered in column (3). All the variables and data sources are described in Appendices A-B. All variables are standardized within the regression sample so that the coefficients in the regression represent the change in SD units of the dependent variable associated with a 1-SD increase in the explanatory variable and are equivalent to Pearson correlation coefficients. The model is described in Appendix C (Model (3b)). Robust standard errors in parentheses *** *p<0.01,* ** *p<0.05,* * *p<0.1*

Table S2D. Country-level analysis. Link between gender gaps in attitudes towards academic effort and gender differences in choices or expectations of specific academic or occupational fields

Panel (i) Attitudes towards trying hard at school (*THI*)

VARIABLES	**Share Males in Tertiary STEM (Absolute)**	**Share Males in Tertiary STEM (Relative)**	**Share Males in Tertiary Humanities (Absolute)**	**Share Males in Tertiary Humanities (Relative)**	**GAP intentions to pursue math studies and careers**	**GAP expectations health occupation (G-B)**

					(B-G)	
**Gap *THI***	**-0.196**	**0.301****	**-0.423****	**-0.201**	**0.247****	**0.339****
	(0.207)	(0.128)	(0.177)	(0.226)	(0.113)	(0.143)
Constant	0.0385	-0.0631	0.0731	0.0420	3.66e-09	-3.24e-09
	(0.166)	(0.168)	(0.150)	(0.166)	(0.125)	(0.121)
Observations	40	38	41	38	61	61
R-squared	0.033	0.079	0.153	0.035	0.061	0.115

Panel (ii) Attitudes towards learning activities (*ATTLNACT*)

VARIABLES	**Share Males in Tertiary STEM (Absolute)**	**Share Males in Tertiary STEM (Relative)**	**Share Males in Tertiary Humanities (Absolute)**	**Share Males in Tertiary Humanities (Relative)**	**GAP intentions to pursue math studies and careers (B-G)**	**GAP expectations health occupation (G-B)**
**Gap *ATTLNACT***	**-0.130**	**0.347*****	**-0.341***	**-0.159**	**0.280*****	**0.347****
	(0.197)	(0.114)	(0.169)	(0.221)	(0.105)	(0.147)
Constant	0.0278	-0.0866	0.0571	0.0395	3.03e-09	-4.02e-09
	(0.168)	(0.160)	(0.155)	(0.174)	(0.124)	(0.121)
Observations	40	38	41	38	61	61
R-squared	0.015	0.110	0.113	0.023	0.078	0.121

Notes. The table presents estimates at the country level of linear regressions of gender gaps in choices or expectations of academic fields and occupations on gender gaps in attitudes towards academic effort. Attitudes towards academic effort are captured in Panel (i) by students' agreement with PISA statement *'Trying hard at school is important'* (*THI*) and in Panel (ii) by the PISA index of attitudes towards learning activities (*ATTLNACT*). Positive gender gaps correspond to more positive attitudes towards academic effort among girls than among boys. Column (1) considers the share of males among students enrolled in tertiary education in STEM and column (3) considers the share of males among students enrolled in tertiary education in Arts and Humanities. Col. (2) and (4) consider the same outcomes but relative to the share of males in tertiary education in the same country. Column (5) considers the gender gap (Boys-Girls) in intentions to pursue math studies and careers. Column (6) considers the gender gap (Girls-Boys) in the expectation to work in health-related fields. All the variables and data sources are described in Appendices A-B. All variables are standardized within the regression sample so that the coefficients in the regression represent the change in SD units of the dependent variable associated with a 1-SD increase in the explanatory variable and are equivalent to Pearson correlation coefficients. The model is described in Appendix C (Model (3b)). Robust standard errors in parentheses *** $p<0.01$, ** $p<0.05$, * $p<0.1$

Table S3 Relation between gender differences in attitudes towards academic effort and gender beliefs and norms

Table S3A. Relation between attitudes towards academic effort and growth mindset or attribution of failure to lack of talent. Individual level analysis

	THI	*ATTLNACT*
**GIRLS**		
Growth Mindset	0.0601***	0.0681***
	(0.00266)	(0.00270)
Fear lacking talent	0.0223***	0.0252***
	(0.00268)	(0.00273)
**BOYS**		
Growth Mindset	0.0624***	0.0693***
	(0.00290)	(0.00290)
Fear lacking talent	0.0124***	0.00638*
	(0.00331)	(0.00334)
**ALL**		
Girl*Growth mindset	-0.00275	-0.00162
	(0.00390)	(0.00400)
Girl*Fear lacking talent	0.00947**	0.0182***
	(0.00433)	(0.00411)

Notes. The table presents estimates of linear regressions at the individual level of indicators of attitudes towards academic effort on a growth mindset indicator and an indicator of fear of lacking talent when facing failure. Attitudes towards academic effort are captured in the first column by students' agreement with PISA statement *'Trying hard at school is important'* (*THI*) and in the second column by the PISA index of attitudes towards learning activities (*ATTLNACT*). Higher *THI* or *ATTLNACT* values correspond to more positive attitudes towards academic effort. The indicator of growth mindset is based on students' (dis)agreement with the statement "*Your intelligence is something about you that you can't change very much*" and that of fear of lacking of talent when facing failure on students' agreement with the statement "*When I am failing, I am afraid that I might not have enough talent*". Results are presented for girls only, for boys only, and for the gender differences. Description of all variables are provided in Appendices A-B. All variables are standardized within country-year. Estimation uses senate weights (student weights normalized to sum to one within country-year on the regression sample) and BRR/Fay replicate weights. The model is described in Appendix C (Model (3a)). Standard errors in parentheses *** $p<0.01$, ** $p<0.05$, * $p<0.1$

Table S3B. Relation between gender gaps in attitudes towards academic effort, gender gaps in ability beliefs/parental support and gender norms. Country-level analysis

Panel (i) Attitudes towards trying hard at school (*THI*)

VARIABLES	Dependent variable is gender gap in THI					
**Gap growth mindset**	**0.419*** **					
	(0.0999)					
**Gap fear of lacking talent**		**0.297** **				
		(0.140)				
**Gap parental support**			**0.279***			
			(0.141)			
**Implicit career family stereotypes**				**0.434*** **		
				(0.123)		
**Communality stereotypes**					**0.437*** **	
					(0.134)	
**Agency stereotypes**						**-0.0938**
						(0.134)
Constant	-0.0430	-0.0548	-0.0255	-0.00665	-0.00669	0.0141
	(0.111)	(0.119)	(0.127)	(0.123)	(0.130)	(0.152)
OBS	60	59	59	53	42	42
R-squared	0.195	0.097	0.077	0.194	0.217	0.001

Panel (ii) Attitudes towards learning activities (*ATTLNACT*)

VARIABLES	Dependent variable is gender gap in ATTLNACT			
**Gap growth mindset**	**0.429*** **			
	(0.108)			
**Gap fear of lacking talent**		**0.256** **		
		(0.124)		
**Gap parental support**			**0.282** **	
			(0.133)	
**Implicit career family stereotypes**				**0.450*** **
				(0.144)

**Communality stereotypes**					**0.446*****	
					(0.141)	
**Agency stereotypes**						**-0.0294**
						(0.142)
Constant	-0.0339	-0.0348	-0.0110	-0.0170	0.0141	0.0141
	(0.114)	(0.124)	(0.128)	(0.129)	(0.135)	(0.152)
OBS	60	59	59	53	42	42
R-squared	0.194	0.068	0.077	0.189	0.210	0.001

Notes. The table presents estimates at the country level of linear regressions of gender gaps in attitudes towards academic effort on gender differences in ability beliefs (growth mindset and fear of lacking talent when facing failure) and parental support as well as gender norms. Attitudes towards academic effort are captured in Panel (i) by students' agreement with PISA statement *'Trying hard at school is important'* (*THI*) and in Panel (ii) by the PISA index of attitudes towards learning activities (*ATTLNACT*). Positive gender gaps correspond to more positive attitudes towards academic effort among girls than among boys. The first row considers country-level gender differences (Girls-Boys) in growth mindset, the second row considers country level gender differences (Girls-Boys) in fear of lacking talent when facing failure, and the third row considers country level gender differences (Girls-Boys) in perceived parental academic support, all based on PISA data. Country level implicit gender stereotypes about career and family among males, based on data from Project Implicit (3), are considered in the fourth row and country-level prescriptive gender stereotypes about communality and agency, based on data from (7), are considered in the last rows. All indicators of gender gaps and gender norms are standardized on the regression sample. All the variables and data sources are described in Appendices A-B. The model is described in Appendix C (Model (3a)). Robust standard errors in parentheses *** $p<0.01$, ** $p<0.05$, * $p<0.1$

Appendix E: Robustness to Spatial Dependence: GS2SLS spatial error models

We tested for spatial dependence in the country-level gender gaps in effort-related attitudes using Moran's I with a row-standardized inverse-distance weights matrix. Results indicated significant spatial autocorrelation in the full sample, suggesting that geographically proximate countries tend to display similar gender gaps. When excluding East and Southeast Asian countries (the cultural group considered in Section 1), spatial dependence becomes non-significant, indicating that most of the spatial autocorrelation originates from this small, contiguous regional cluster of culturally similar countries.

As a robustness check, we re-estimated the main country-level regressions (reported in Appendix D) using generalized spatial two-stage least squares (GS2SLS) spatial error models that explicitly account for cross-country spatial dependence. We used a row-normalized inverse-distance weights matrix (W) and report both OLS and GS2SLS estimates on common samples. These models confirm that the associations between gender gaps in effort-related attitudes and country-level indicators—of development, academic performance, educational attainment, and cultural attitudes—remain robust when accounting for spatial autocorrelation. Spatial error terms are significant in some specifications, indicating regional clustering in residuals, but the main coefficients remain stable (and often only slightly attenuated), suggesting that the observed relationships are not artifacts of spatial clustering.

Note besides that all OLS estimates remain significant, showing the robustness of our results to different samples of countries.

Global Moran's I on the dependent variable

We tested for spatial dependence in the country-level gender gaps in effort-related attitudes using Moran's I with a row-standardized inverse-distance weights matrix.
Results indicated significant spatial autocorrelation on the whole sample ($\chi^2(1)=10.5$, $p=0.001$ for *THI* and $\chi^2(1)=12.9$, $p=0$ for *ATTLNACT*, N=61).
When excluding the cultural group of East and Southeast Asian countries (Hong Kong, Indonesia, Japan, Korea, Macao, Malaysia, Singapore, Chinese Taipei, Thailand, Vietnam), spatial dependence becomes non-significant ($p=0.11$ for *THI* and $p=0.28$ for *ATTLNACT*, N=51).

Panel i) Attitudes towards trying hard at school (*THI*)

Sample	N	Tested model	Spatial weights (W)	$\chi^2(1)$	p-value	5% sig.
**Full sample**	61	Residuals of reg gap_*THI* (intercept only)	inverse-distance, row-normalized	10.49	**0.0012**	YES
**Excl. East/Southeast Asia (10)**	51	Same	Same	2.57	**0.11**	NO

Panel (ii): Attitudes towards learning activities (*ATTLNACT*)

Sample	N	Tested model	Spatial weights (W)	$\chi^2(1)$	p-value	5% sig.
**Full sample**	61	Residuals of reg gap_*ATTLNACT* (intercept only)	inverse-distance, row-normalized	12.88	**0.0003**	YES
**Excl. East/Southeast Asia (10)**	51	Same	Same	1.16	**0.28**	NO

Table S1D using spatial error models

In the following Tables we consider the robustness to control for spatial dependencies of the link shown in Table S1D between gender gaps in attitudes towards academic effort and country indicators of economic development or gender equality. We provide OLS and GS2SLS estimates controlling for spatial autocorrelation across countries.

The first column provides the results of OLS regressions on the common 51-country sample which is the analog of Table S1D on this restricted sample. Results are very similar: all five development indicators are positively and significantly associated with the gender gap in effort-related attitudes: for *THI*, a 1-SD increase in ESCS/HDI/lnGDP/GGI/Individualism is linked to roughly 0.41–0.54 SD larger gaps. This means that results of Table S1D are robust to a different sample of countries.
Re-estimating on the same sample with a spatial error model (GS2SLS) confirms these patterns. All estimates remain positive and significant once spatial dependence is modeled. Spatial error terms are significant for HDI/lnGDP/GGI/Individualism, indicating regional clustering in the residuals, but the key coefficients persist (often slightly attenuated), suggesting the relationships are not artifacts of spatial clustering.

Panel i) Attitudes towards trying hard at school (*THI*)

	**OLS**	**Spatial error model (GS2SLS)** *Dependent variable is Gender gap in THI*				
**ESCS**	**0.539*****	**0.513*****				
	(0.0945)	(0.124)				
**HDI**	**0.411****		**0.328****			
	(0.166)		(0.135)			
**lnGDP**	**0.433*****			**0.363*****		
	(0.141)			(0.134)		
**GGI**	**0.430****				**0.363*****	
	(0.206)				(0.133)	
**Indiv.**	**0.423*****					**0.336****
	(0.128)					(0.135)
Spatial Error		0.595	0.944**	0.995**	0.868**	1.144**
		(0.458)	(0.420)	(0.419)	(0.386)	(0.457)
Obs.		51	51	51	51	51
pseudo R-squared		0.29	0.169	0.187	0.185	0.179

Panel (ii) Attitudes towards learning activities (*ATTLNACT*)

	**OLS**	**Spatial error model (GS2SLS)** *Dependent variable is Gender gap in ATTLNACT*				
**ESCS**	**0.503*****	**0.484*****				
	(0.116)	(0.125)				
**HDI**	**0.408****		**0.338****			
	(0.156)		(0.135)			
**lnGDP**	**0.419*****			**0.354*****		
	(0.136)			(0.135)		
**GGI**	**0.427****				**0.367*****	
	(0.173)				(0.133)	
**Indiv.**	**0.418*****					**0.354*****
	(0.135)					(0.134)

Spatial Error		0.507	0.806	1.120**	0.680	0.832
		(0.671)	(0.604)	(0.441)	(0.479)	(0.696)
Obs.		51	51	51	51	51
pseudo R-squared		0.254	0.167	0.176	0.182	0.175

Notes. See the Notes of Table S1D

Table S2B using spatial error models

In the following Tables we consider the robustness to control for spatial dependencies of the link shown in Table S2B between gender gaps in attitudes towards academic effort and country indicators of academic performance.
The last row provides the results of OLS regressions on the full 61-country sample. When accounting for spatial dependence, spatial error models on the full 61-country sample yield very similar magnitudes, and all coefficients remain significant: a larger gender gap in *THI* or *ATTLNACT* is associated with wider performance gaps overall and by subject and with more male over-representation among low performers, both overall and in reading. The spatial error terms are significant—indicating regional clustering in residuals—but the associations hold after controlling for these unobserved spatial components.

Panel i) Attitudes towards trying hard at school (*THI*)

	Spatial error model (GS2SLS) **Dependent variable is Gender gap (G-B) in ...**					
VARIABLES	**Gen. Score**	**Math score**	**Reading score**	**Science score**	**Likelihood low performer**	**Likelihood low perf. in reading**
**Gap *THI***	**0.395*** **	**0.349*** **	**0.401*** **	**0.358*** **	**-0.350*** **	**-0.406*** **
	(0.107)	(0.110)	(0.108)	(0.109)	(0.114)	(0.115)
Spatial Error	1.599***	1.621***	1.607***	1.617***	1.351***	1.134***
	(0.308)	(0.370)	(0.334)	(0.300)	(0.292)	(0.257)
Obs.	61	61	61	61	61	61
R-squared	0.188	0.123	0.214	0.163	0.166	0.224
OLS	**0.433*** **	**0.350** **	**0.463*** **	**0.403** **	**-0.407*** **	**-0.473*** **
	(0.151)	(0.143)	(0.144)	(0.161)	(0.151)	(0.139)

Panel (ii) Attitudes towards learning activities (*ATTLNACT*)

VARIABLES	Gen. Score	Math score	Reading score	Science score	Likelihood low performers	Likelihood low perf. in reading
**Gap** *ATTLNACT*	**0.385*** **	**0.365*** **	**0.360*** **	**0.366*** **	**-0.358*** **	**-0.393*** **
	(0.109)	(0.109)	(0.112)	(0.109)	(0.115)	(0.117)
Spatial Error	1.655***	1.726***	1.589***	1.686***	1.443***	1.191***
	(0.303)	(0.395)	(0.325)	(0.294)	(0.291)	(0.283)
Obs.	61	61	61	61	61	61
R-squared	0.147	0.093	0.174	0.130	0.141	0.204
OLS	**0.384*** **	**0.305** **	**0.417*** **	**0.360** **	**-0.376*** **	**-0.451*** **
	(0.141)	(0.144)	(0.140)	(0.142)	(0.137)	(0.127)

Notes. See the Notes of Table S2B

Table S2C using spatial error models

In the following Tables we consider the robustness to control for spatial dependencies of the link shown in Table S2C between gender gaps in attitudes towards academic effort and country indicators of male academic underachievement.

The last row provides the results of OLS regressions on the common 39-country sample, which is the analog of Table S2C on this restricted sample. The consideration of a smaller sample does not alter the results: countries with larger gender gaps in effort show fewer men in tertiary education and a higher female-to-male ratio in expected years of schooling.

On the same country sample, spatial error models (GS2SLS) yield very similar magnitudes. We find no evidence of residual spatial autocorrelation.

Panel (i) Attitudes towards trying hard at school (*THI*)

VARIABLES	**Male enrollment in tertiary education (2)**	**Gender Gap in expected years of schooling**
**Gender gap *THI***	**-0.466*** **	**0.399*** **
	(0.140)	(0.146)
Spatial Error	0.545	1.305
	(0.988)	(1.152)
Observations	39	39
Pseudo R-squared	0.227	0.165
OLS	**-0.476** **	**0.406*** **
	(0.190)	(0.141)

Panel (ii) Attitudes towards learning activities (*ATTLNACT*)

VARIABLES	**Male enrollment in tertiary education (2)**	**Gender Gap in expected years of schooling**
**Gender gap *ATTLNACT***	**-0.461*** **	**0.299** **
	(0.140)	(0.151)
Spatial Error	0.447	1.398
	(1.202)	(1.188)
Observations	39	39
R-squared	0.225	0.098
OLS	**-0.474** **	**0.313* **
	(0.189)	(0.173)

Notes. See the Notes of Table S2C

Table S2D using spatial error models

In OLS on the common sample, restricted to 38 countries, results are similar although slightly weaker than on the larger 61-countries sample: larger gender gaps in the gender gap in *THI* are associated with fewer men in tertiary arts & humanities, and this is not the case for STEM, where the relation is not significant in absolute terms and positive in relative terms. Larger gender gaps in *THI* are also associated with a larger female–male gap in health-occupation expectations. Note that in this smaller sample the relation with the gender gap in intentions to pursue math studies and careers is positive but does not reach significance.

Spatial error models (GS2SLS) on the same sample confirm these relationships. With significant regional clustering, the coefficients are in some cases attenuated and sometimes reinforced (for health occupations). Overall, the patterns are not artifacts of spatial clustering.

Panel (i) Attitudes towards trying hard at school (*THI*)

VARIABLES	**Share Males in Tertiary STEM (Absolute)**	**Share Males in Tertiary STEM (Relative)**	**Share Males in Tertiary Humanities (Absolute)**	**Share Males in Tertiary Humanities (Relative)**	**GAP expectations health occupation (G-B)**
**Gap *THI***	**-0.191**	**0.253***	**-0.281****	**-0.0444**	**0.445*****
	(0.154)	(0.152)	(0.127)	(0.140)	(0.138)
Spatial Error	0.176	1.530***	1.525***	2.588***	0.852***
	(1.058)	(0.294)	(0.210)	(0.768)	(0.115)
Observations	38	38	38	38	38
R-squared	0.038	0.079	0.158	0.035	0.141
**OLS**	**-0.194**	**0.280****	**-0.397****	**-0.187**	**0.375****
	(0.192)	(0.119)	(0.170)	(0.210)	(0.161)

Panel (ii) Attitudes towards learning activities (*ATTLNACT*)

VARIABLES	**Share Males in Tertiary STEM (Absolute)**	**Share Males in Tertiary STEM (Relative)**	**Share Males in Tertiary Humanities (Absolute)**	**Share Males in Tertiary Humanities (Relative)**	**GAP expectations health occupation (G-B)**
**Gap *ATTLNACT***	**-0.153**	**0.319****	**-0.224***	**-0.00558**	**0.384*****
	(0.158)	(0.151)	(0.126)	(0.136)	(0.140)
Spatial Error	0.0999	0.437	1.517***	2.508***	0.871***
	(1.086)	(1.026)	(0.213)	(0.702)	(0.102)
Observations	38	38	38	38	38
R-squared	0.02	0.110	0.13	0.02	0.10
OLS	**-0.142**	**0.332*****	**-0.361****	**-0.151**	**0.315**
	(0.190)	(0.109)	(0.166)	(0.211)	(0.198)

Notes. See the Notes of Table S2D

Table S3B using spatial error models

In the following Tables we consider the robustness to control for spatial dependencies of the link shown in Table S3B between gender gaps in attitudes towards academic effort and cultural indicators such as gender gaps in growth mindset, or gender stereotypes.

In OLS, on the common 37-country sample, results concerning gaps in growth mindset, in implicit stereotypes about career and family and in stereotypes about women's communality are very similar to those obtained in Table S3B on the full sample. Note that the estimate for the gender gap in parental support (β=0.22) did not reach significance on this restricted sample.

Re-estimating on the common 37-country sample with spatial error models (GS2SLS) yields very similar—and in some cases slightly larger—coefficients for the four variables, confirming robustness to accounting for spatially correlated unobservables.

Panel (i) Attitudes towards trying hard at school (*THI*)

	**OLS**	**GLS MODELS**		
**Gender gap Growth mindset**	**0.516*****	**0.492*****		
	(0.124)	(0.138)		
**Implicit stereotypes (Career-Family, Males)**	**0.442****		**0.425*****	
	(0.171)		(0.155)	
**Stereotypes about communality**	**0.546*****			**0.525*****
	(0.152)			(0.133)
Spatial Error		1.363*	1.901	1.373***
		(0.778)	(1.469)	(0.504)
Obs.	38	38	38	38
R-squared		0.266	0.195	0.298

Panel (ii) Attitudes towards learning activities (*ATTLNACT*)

	**OLS**	**GLS MODELS**
**Gender gap Growth mindset**	**0.428****	**0.413*****
	(0.168)	(0.147)

**Implicit stereotypes (Career-Family, Males)**	**0.411****		**0.416*****	
	(0.183)		(0.145)	
**Stereotypes about communality**	**0.520*****			**0.508*****
	(0.148)			(0.138)
Spatial Error		0.455	1.244	0.325
		(0.922)	(1.514)	(0.739)
Obs.	38	38	38	38
R-squared		0.183	0.169	0.27

Notes. See the Notes of Table S3B

SI References

1. G. H. Hofstede, G. J. Hofstede, M. Minkov, *Cultures and organizations: software of the mind: intercultural cooperation and its importance for survival*, 3rd ed (McGraw-Hill, 2010).

2. C. L. Fincher, R. Thornhill, Parasite-stress promotes in-group assortative sociality: The cases of strong family ties and heightened religiosity. *Behav Brain Sci* **35**, 61–79 (2012).

3. B. A. Nosek, M. R. Banaji, A. G. Greenwald, Harvesting implicit group attitudes and beliefs from a demonstration web site. *Group Dynamics: Theory, Research, and Practice* **6**, 101–115 (2002).

4. A. G. Greenwald, D. E. McGhee, J. L. Schwartz, Measuring individual differences in implicit cognition: the implicit association test. *J Pers Soc Psychol* **74**, 1464–1480 (1998).

5. C. Napp, Gender Stereotypes About Career and Family Are Stronger in More Economically Developed Countries and Can Explain the Gender Equality Paradox. *Personality and Social Psychology Bulletin* (2024). https://doi.org/10.1177/01461672241286084.

6. N. Kosakowska-Berezecka, *et al.*, Towards Gender Harmony Dataset: Gender Beliefs and Gender Stereotypes in 62 Countries. *Sci Data* **11**, 392 (2024).

7. N. Kosakowska-Berezecka, *et al.*, Does Culture Moderate Gender Stereotypes? Individualism Predicts Communal (but Not Agentic) Prescriptions for Men Across 62 Nations. *Social Psychological and Personality Science* (2024). https://doi.org/10.1177/19485506231221913.

8. F. Avvisati, F. Keslair, REPEST: Stata module to run estimations with weighted replicate samples and plausible values. (2024). Deposited 11 December 2024.
